# Supplementary material for: Migraine patients visiting Chinese medicine hospital: Protocol for a prospective, registry-based, real-world observational cohort study
Source: PLoS One. 2022 Mar 15;17(3):e0265137. doi: 10.1371/journal.pone.0265137 (PMC8923465; doi:10.1371/journal.pone.0265137)
Supplement: S1 Checklist — (DOCX) [file pone.0265137.s001.docx]

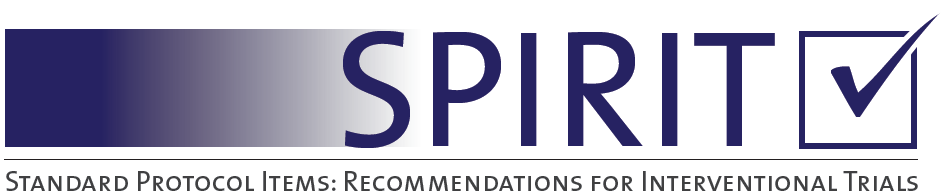


SPIRIT 2013 Checklist: Recommended items to address in a clinical trial protocol and related documents*

| Section/item | ItemNo | Description |
| --- | --- | --- |
| **Administrative information** | | |
| Title | 1 | Descriptive title identifying the study design, population, interventions, and, if applicable, trial acronym. Page 1, Line 1&2 |
| Trial registration | 2a | Trial identifier and registry name. If not yet registered, name of intended registry Page 3, Line 40; Page 7, Line 117. |
|  | 2b | All items from the World Health Organization Trial Registration Data Set All WHO Trial Registration Data requirements are met with the trial’s registration in the ClinicalTrials.gov. Trial registration information is found on Page 3, Line 40; Page 7, Line 117. |
| Protocol version | 3 | Date and version identifier Page 7, Line 113. |
| Funding | 4 | Sources and types of financial, material, and other support Page 19, Lines 322-323. |
| Roles and responsibilities | 5a | Names, affiliations, and roles of protocol contributors Page 19, Lines 315-320. |
|  | 5b | Name and contact information for the trial sponsor NA. |
|  | 5c | Role of study sponsor and funders, if any, in study design; collection, management, analysis, and interpretation of data; writing of the report; and the decision to submit the report for publication, including whether they will have ultimate authority over any of these activities The sponsor and funders did not involve in any of these activities. |
|  | 5d | Composition, roles, and responsibilities of the coordinating centre, steering committee, endpoint adjudication committee, data management team, and other individuals or groups overseeing the trial, if applicable (see Item 21a for data monitoring committee) This is an observational study, no intervention was designed, and no data monitoring committee was |
| Introduction |  |  |
| Background and rationale | 6a | Description of research question and justification for undertaking the trial, including summary of relevant studies (published and unpublished) examining benefits and harms for each intervention Pages 4-6, Lines 42-92. |
|  | 6b | Explanation for choice of comparators This is an observational cohort study and no comparators were used. |
| Objectives | 7 | Specific objectives or hypotheses Pages 6, Lines 93-99. |
| Trial design | 8 | Description of trial design including type of trial (eg, parallel group, crossover, factorial, single group), allocation ratio, and framework (eg, superiority, equivalence, noninferiority, exploratory) Page 7, Lines 102-103. |
| Methods: Participants, interventions, and outcomes | | |
| Study setting | 9 | Description of study settings (eg, community clinic, academic hospital) and list of countries where data will be collected. Reference to where list of study sites can be obtained Page 7, Lines 102-103. |
| Eligibility criteria | 10 | Inclusion and exclusion criteria for participants. If applicable, eligibility criteria for study centres and individuals who will perform the interventions (eg, surgeons, psychotherapists) Pages 8, Lines 121-132. |
| 136Interventions | 11a | Interventions for each group with sufficient detail to allow replication, including how and when they will be administered Page 8, Lines 134-138. But the interventions are prescribed by the Chinese medicine practitioners, totally independent of the study design. |
|  | 11b | Criteria for discontinuing or modifying allocated interventions for a given trial participant (eg, drug dose change in response to harms, participant request, or improving/worsening disease) Page 9, Line 133-136. |
|  | 11c | Strategies to improve adherence to intervention protocols, and any procedures for monitoring adherence (eg, drug tablet return, laboratory tests) The interventions are not compulsory designed, so is the adherence. |
|  | 11d | Relevant concomitant care and interventions that are permitted or prohibited during the trial Page 8, Lines137-138. |
| Outcomes | 12 | Primary, secondary, and other outcomes, including the specific measurement variable (eg, systolic blood pressure), analysis metric (eg, change from baseline, final value, time to event), method of aggregation (eg, median, proportion), and time point for each outcome. Explanation of the clinical relevance of chosen efficacy and harm outcomes is strongly recommended Pages 9-12, Lines 147-209. |
| Participant timeline | 13 | Time schedule of enrolment, interventions (including any run-ins and washouts), assessments, and visits for participants. A schematic diagram is highly recommended (see Figure) Page 13, Figure 1 |
| Sample size | 14 | Estimated number of participants needed to achieve study objectives and how it was determined, including clinical and statistical assumptions supporting any sample size calculations Page 14, Line 216-222. Sample size calculation is not required since there are no pre-determined hypotheses to be tested. |
| Recruitment | 15 | Strategies for achieving adequate participant enrolment to reach target sample size This is a registry-based cohort study, the participants are consecutive recruited within a specific time range. |
| **Methods: Assignment of interventions (for controlled trials)** | | |
| Allocation: |  |  |
| Sequence generation | 16a X | Method of generating the allocation sequence (eg, computer-generated random numbers), and list of any factors for stratification.  To reduce predictability of a random sequence, details of any planned restriction (eg, blocking) should be provided in a separate document that is unavailable to those who enrol participants or assign interventions This is a registry-based observational study rather than a RCT. The participants are consecutively recruited. Allocation sequence is not required in this study. |
| Allocation concealment mechanism | 16b | Mechanism of implementing the allocation sequence (eg, central telephone; sequentially numbered, opaque, sealed envelopes), describing any steps to conceal the sequence until interventions are assigned This is a registry-based observational study rather than a RCT. The participants are consecutively recruited. Allocation sequence concealment is not required in this study. |
| Implementation | 16c | Who will generate the allocation sequence, who will enrol participants, and who will assign participants to interventions Same as 16a and 16b. |
| Blinding (masking) | 17a | Who will be blinded after assignment to interventions (eg, trial participants, care providers, outcome assessors, data analysts), and how This is a real-world study, the interventions are prescribed by the clinicians based on real conditions. Blinding is not required in this study. |
|  | 17b | If blinded, circumstances under which unblinding is permissible, and procedure for revealing a participant’s allocated intervention during the trial This is a real-world study, the interventions are prescribed by the clinicians based on real conditions. Blinding is not required in this study. |
| **Methods: Data collection, management, and analysis** | | |
| Data collection methods | 18a | Plans for assessment and collection of outcome, baseline, and other trial data, including any related processes to promote data quality (eg, duplicate measurements, training of assessors) and a description of study instruments (eg, questionnaires, laboratory tests) along with their reliability and validity, if known. Reference to where data collection forms can be found, if not in the protocol Pages 9-13, Line 148-214. Detailed CRF and migraine diary could be found in S1 and S2 files. |
|  | 18b | Plans to promote participant retention and complete follow-up, including list of any outcome data to be collected for participants who discontinue or deviate from intervention protocols Page 12, Lines 207-209. |
| Data management | 19 | Plans for data entry, coding, security, and storage, including any related processes to promote data quality (eg, double data entry; range checks for data values). Reference to where details of data management procedures can be found, if not in the protocol Page 15, Lines 235-243. |
| Statistical methods | 20a | Statistical methods for analysing primary and secondary outcomes. Reference to where other details of the statistical analysis plan can be found, if not in the protocol Pages 15-16, Lines 245-262. |
|  | 20b | Methods for any additional analyses (eg, subgroup and adjusted analyses) Page 15, Line 247-249. |
|  | 20c | Definition of analysis population relating to protocol non-adherence (eg, as randomised analysis), and any statistical methods to handle missing data (eg, multiple imputation) Page 16, Line 261-262. |
| **Methods: Monitoring** | | |
| Data monitoring | 21a | Composition of data monitoring committee (DMC); summary of its role and reporting structure; statement of whether it is independent from the sponsor and competing interests; and reference to where further details about its charter can be found, if not in the protocol. Alternatively, an explanation of why a DMC is not needed This is a real-world, registry-based, observational study without risks, no experimental interventions are used in this study. DMC is not needed in this study. |
|  | 21b | Description of any interim analyses and stopping guidelines, including who will have access to these interim results and make the final decision to terminate the trial No experimental interventions are applied and the item is not applicable in this study. |
| Harms | 22 | Plans for collecting, assessing, reporting, and managing solicited and spontaneously reported adverse events and other unintended effects of trial interventions or trial conduct Page 11, lines 192-194. Page 12, line 205-209 |
| Auditing | 23 | Frequency and procedures for auditing trial conduct, if any, and whether the process will be independent from investigators and the sponsor No audit is proposed in this study, similar reason to 21a. |
| Ethics and dissemination | | |
| Research ethics approval | 24 | Plans for seeking research ethics committee/institutional review board (REC/IRB) approval Page 7, Lines 113-114. |
| Protocol amendments | 25 | Plans for communicating important protocol modifications (eg, changes to eligibility criteria, outcomes, analyses) to relevant parties (eg, investigators, REC/IRBs, trial participants, trial registries, journals, regulators) NA. This observational study is going on well for now, thanks to the well trial design. Currently, no plans for amendments is scheduled. |
| Consent or assent | 26a | Who will obtain informed consent or assent from potential trial participants or authorised surrogates, and how (see Item 32) Page 9, Lines 142-145. |
|  | 26b | Additional consent provisions for collection and use of participant data and biological specimens in ancillary studies, if applicable NA |
| Confidentiality | 27 | How personal information about potential and enrolled participants will be collected, shared, and maintained in order to protect confidentiality before, during, and after the trial Pages 15, Lines 240-243. |
| Declaration of interests | 28 | Financial and other competing interests for principal investigators for the overall trial and each study site Page 19, Lines 324-325. |
| Access to data | 29 | Statement of who will have access to the final trial dataset, and disclosure of contractual agreements that limit such access for investigators Page 15, Lines 242-243. |
| Ancillary and post-trial care | 30 | Provisions, if any, for ancillary and post-trial care, and for compensation to those who suffer harm from trial participation No harms are anticipated in this observational study. |
| Dissemination policy | 31a | Plans for investigators and sponsor to communicate trial results to participants, healthcare professionals, the public, and other relevant groups (eg, via publication, reporting in results databases, or other data sharing arrangements), including any publication restrictions NA. |
|  | 31b | Authorship eligibility guidelines and any intended use of professional writers NA |
|  | 31c | Plans, if any, for granting public access to the full protocol, participant-level dataset, and statistical code NA |
| Appendices |  |  |
| Informed consent materials | 32 | Model consent form and other related documentation given to participants and authorised surrogates. The informed consent letter will be provided if requested. |
| Biological specimens | 33 | Plans for collection, laboratory evaluation, and storage of biological specimens for genetic or molecular analysis in the current trial and for future use in ancillary studies, if applicable. No biological specimen collection is planned in this study since we did not propose any compulsory examination or interventions. |

*It is strongly recommended that this checklist be read in conjunction with the SPIRIT 2013 Explanation & Elaboration for important clarification on the items. Amendments to the protocol should be tracked and dated. The SPIRIT checklist is copyrighted by the SPIRIT Group under the Creative Commons “[Attribution-NonCommercial-NoDerivs 3.0 Unported](http://www.creativecommons.org/licenses/by-nc-nd/3.0/)” license.
