# Supplementary material for: Migraine patients visiting Chinese medicine hospital: Protocol for a prospective, registry-based, real-world observational cohort study
Source: PLoS One. 2022 Mar 15;17(3):e0265137. doi: 10.1371/journal.pone.0265137 (PMC8923465; doi:10.1371/journal.pone.0265137)
Supplement: S1 File — (DOCX) [file pone.0265137.s002.docx]

Questionnaire 1: Demographic and Background Information

| Name |  | Date |  | Telephone No. |  |
| --- | --- | --- | --- | --- | --- |
| Gender | ☐Male ☐Female | Migraine course |  | Age |  |
| (Female only) Migraine attack associated with menstrual cycle? | | | ☐Yes | ☐No | ☐Unclear |
| Any aura symptom preceding headache attack? | | | ☐Yes | ☐No | ☐Unclear |
| Any migraine family history? | | | ☐Yes | ☐No | ☐Unclear |
| What do you concern the most about the coming medical visit? | | | ☐Impact of medicine on migraine | | |
|  |  |  | ☐Impact of the medicine on quality of life | | |
|  |  |  | ☐Impact of the medicine on psychological state | | |
|  |  |  | ☐Impact of the medicine on sleeping quality | | |
|  |  |  | ☐Potential side effects of the medicine | | |
|  |  |  | ☐Potential reduction on the acute medication usage | | |
|  |  |  | ☐Administration method of the medicine | | |
|  |  |  | ☐Acceptance of the medicine | | |
|  |  |  | ☐Treating course | | |
|  |  |  | ☐Frequency of taking the medicine | | |
|  |  |  | ☐Fees | | |
|  |  |  | ☐Others, please indicate: __________________ | | |

Questionnaire 2: Migraine Severity and Medication Usage

Please answer the following questions based on your situations in **the last 4 weeks**.

| 1. What is the migraine attack frequency in the last 4 weeks? (Attacks within 48 hours are defined as one attack) | |  | |
| --- | --- | --- | --- |
| 2. What is the average migraine duration in the last 4 weeks? (hours) | |  | |
| 3. What is the most severe migraine pain in the last 4 weeks? (From 0 to 10, 0 refers to no pain, while 10 refers to most possible severe pain) | |  | |
| 4. What is the average pain in the last 4 weeks? (From 0 to 10, 0 refers to no pain, while 10 refers to most possible severe pain) | |  | |
| 5. How many days did you suffer from migraine in the last 4 weeks? | |  | |
| 6. Have you ever taken any prophylactic medicine for migraine in the last 4 weeks? | | ☐Yes | ☐No |
| 7. Which prophylactic treatment (s) for migraine did you take in the last 4 weeks? (*Skip this item if you did not take any prophylactic medicine*) | ☐Flunarizine ☐Nimodipine | | |
|  | ☐Valproate ☐Topiramate ☐Gabapentin | | |
|  | ☐Metoprolol ☐Propranolol ☐Bisoprolol | | |
|  | ☐Amitriptyline | | |
|  | ☐Candesartan ☐Lisinopril | | |
|  | ☐*Zhengtian* pill ☐*Tianshu* tablet ☐*Duliang* pill ☐Chinese herbal medicine decoction | | |
|  | ☐Acupuncture ☐*Tuina* ☐Moxibustion | | |
|  | ☐Others (please indicate): _________________________________ | | |
| 8. Have you ever taken any analgesics for migraine? | ☐Yes | | ☐No |
| **Please answer Questions 9-15 if your take analgesics for migraine** | | | |
| 9. Which medicine (s) did you take for migraine in the last 4 weeks? | ☐Ibuprofen ☐Diclofenac | | |
|  | ☐Celecoxib ☐Etoricoxib | | |
|  | ☐Acetaminophen (Paracetamols, Tylenol, Panadol, Bufferin) | | |
|  | ☐Naproxen | | |
|  | ☐Aspirin | | |
|  | ☐Combination such as Paracetamol Caffeine Aspirin Powder | | |
|  | ☐Sumatriptan ☐Zolmitriptan | | |
|  | ☐Naratriptan ☐Rizatriptan | | |
|  | ☐Ergotamine Tartrate ☐Dihydroergotamine | | |
|  | ☐CGRP: Telcagepant | | |
|  | ☐Others (please indicate): _________________________________ | | |
| 10. What is the average dosage of analgesics you took per time in the last 4 weeks? |  | | |
| 11. How many analgesics did you take in total in the last 4 weeks? |  | | |
| 12. How many days out of the last 4 weeks did you take analgesics? |  | | |
| 13. Did you get professional advice regarding the analgesics for migraine? | ☐Yes, advised by my doctors | | |
|  | ☐Yes, advised by other doctors | | |
|  | ☐Yes, advised by the pharmacist | | |
|  | ☐No | | |
| 14. Did you suffer from any adverse effect in the last 4 weeks? | ☐Yes (please indicate): _____________________________________ | | |
|  | ☐No | | |
| 15. Did you strictly follow the instructions when taking the analgesics? | ☐Yes, I followed the instructions strictly. | | |
|  | ☐No, I only read the instructions but failed to follow them strictly. | | |
|  | ☐No, I neither read the instructions nor followed them. | | |
| **Other question (s)** | | | |
| 16. Did you take any other medicines apart from those for migraine? | ☐Yes (please indicate): __________________________________ | | |
|  | ☐No | | |
| 17. How do you like your comments for the treatment? (**Follow-up visits only**) | ☐Very satisfied | | |
|  | ☐Much satisfied | | |
|  | ☐Satisfies | | |
|  | ☐A bit dissatisfied | | |
|  | ☐Very dissatisfied | | |

Questionnaire 3: Migraine Specific Quality of Life Questionnaire (MSQ)

Please select only one answer for each question. You should answer every question. While answering the following questions, please think about all migraine attacks you may have had in **the past 4 weeks**.

| Items | Never | Rarely | Some of the time | A good bit of the time | Most of the time | All of the time |
| --- | --- | --- | --- | --- | --- | --- |
| 1. In the past 4 weeks, how often have migraines interfered with how well you dealt with family, friends and others who are close to you? | ☐ | ☐ | ☐ | ☐ | ☐ | ☐ |
| 2. In the past 4 weeks, how often have migraines interfered with your leisure time activities, such as reading or exercising? | ☐ | ☐ | ☐ | ☐ | ☐ | ☐ |
| 3. In the past 4 weeks, how often have you had difficulty in performing work or daily activities because of migraine symptoms? | ☐ | ☐ | ☐ | ☐ | ☐ | ☐ |
| 4. In the past 4 weeks, how often have migraines kept you from getting as much done at work or at home? | ☐ | ☐ | ☐ | ☐ | ☐ | ☐ |
| 5. In the past 4 weeks, how often have migraines limited your ability to concentrate on work or daily activities? | ☐ | ☐ | ☐ | ☐ | ☐ | ☐ |
| 6. In the past 4 weeks, how often have migraines left you too tired to do work or daily activities? | ☐ | ☐ | ☐ | ☐ | ☐ | ☐ |
| 7. In the past 4 weeks, how often have migraines limited the number of days you have felt energetic? | ☐ | ☐ | ☐ | ☐ | ☐ | ☐ |
| 8. In the past 4 weeks, how often have you had to cancel work or daily activities because of your migraines? | ☐ | ☐ | ☐ | ☐ | ☐ | ☐ |
| 9. In the past 4 weeks, how often did you need help in handling routine tasks such as every day household chores, doing necessary business, shopping, or caring for others, because of your migraines? | ☐ | ☐ | ☐ | ☐ | ☐ | ☐ |
| 10. In the past 4 weeks, how often did you have to stop work or daily activities to deal with migraine symptoms? | ☐ | ☐ | ☐ | ☐ | ☐ | ☐ |
| 11. In the past 4 weeks, how often were you not able to go to social activities such as parties, dinner with friends, because of your migraines? | ☐ | ☐ | ☐ | ☐ | ☐ | ☐ |
| 12. In the past 4 weeks, how often have you felt fed up or frustrated because of your migraines? | ☐ | ☐ | ☐ | ☐ | ☐ | ☐ |
| 13. In the past 4 weeks, how often have you felt like you were a burden on others because of your migraines? | ☐ | ☐ | ☐ | ☐ | ☐ | ☐ |
| 14. In the past 4 weeks, how often have you been afraid of letting others down because of your migraines? | ☐ | ☐ | ☐ | ☐ | ☐ | ☐ |

Questionnaire 4: Generalized Anxiety Disorder 7-item scale (GAD-7)

Over **the last 2 weeks**, how often have you been bothered by the following problems?

|  | Not at all sure | Several days | Over half the days | Nearly every day |
| --- | --- | --- | --- | --- |
| 1. Feeling nervous, anxious, or on edge |  |  |  |  |
| 2. Not being able to stop or control worrying |  |  |  |  |
| 3. Worrying too much about different things |  |  |  |  |
| 4. Trouble relaxing |  |  |  |  |
| 5. Being so restless that it's hard to sit still |  |  |  |  |
| 6. Becoming easily annoyed or irritable |  |  |  |  |
| 7. Feeling afraid as if something awful might happen |  |  |  |  |

Questionnaire 5: Patient Health Questionnaire-9 (PHQ-9)

Over **the past 2 weeks**, how often have you been bothered by any of the following problems?

|  | | | Not at all | Several days | | More than half the days | Nearly every day |
| --- | --- | --- | --- | --- | --- | --- | --- |
| 1. Little interest or pleasure in doing things | | |  |  | |  |  |
| 2. Feeling down, depressed, or hopeless | | |  |  | |  |  |
| 3. Trouble falling or staying asleep, or sleeping too much | | |  |  | |  |  |
| 4. Feeling tired or having little energy | | |  |  | |  |  |
| 5. Poor appetite or overeating | | |  |  | |  |  |
| 6. Feeling bad about self - or that you are a failure or have let yourself or your family down | | |  |  | |  |  |
| 7. Trouble concentrating on things, such as reading the newspaper or watching television | | |  |  | |  |  |
| 8. Moving or speaking so slowly that other could have noticed? Or the opposite- being so fidgety or restless that you have been moving around a lot more than usual | | |  |  | |  |  |
| 9. Thoughts that you would be better off dead, or of hurting yourself in some way | | |  |  | |  |  |
| If you checked off any problems, how difficult have these problems made it for you to do your work, take care of things at home, or get along with other people? | | | | | | | |
| Not difficult at all | Somewhat difficult | Very difficult | | | Extremely difficult | | |
| ☐ | ☐ | ☐ | | | ☐ | | |

Questionnaire 6: Insomnia Severity Index (ISI)

For each question below, please circle the number corresponding most accurately to your sleep patterns in the **LAST 2 WEEKS**.

For the first three questions, please rate the SEVERITY of your sleep difficulties.

1. Difficulty falling asleep:

| None | Mild | Moderate | Severe | Very Severe |
| --- | --- | --- | --- | --- |
| 0 | 1 | 2 | 3 | 4 |

2. Difficulty staying asleep:

| None | Mild | Moderate | Severe | Very Severe |
| --- | --- | --- | --- | --- |
| 0 | 1 | 2 | 3 | 4 |

3. Problem waking up too early in the morning:

| None | Mild | Moderate | Severe | Very Severe |
| --- | --- | --- | --- | --- |
| 0 | 1 | 2 | 3 | 4 |

4. How SATISFIED/dissatisfied are you with your current sleep pattern?

| Very Satisfied | Satisfied | Neutral | Dissatisfied | Very Dissatisfied |
| --- | --- | --- | --- | --- |
| 0 | 1 | 2 | 3 | 4 |

5. To what extent do you consider your sleep problem to INTERFERE with your daily functioning (e.g. daytime fatigue, ability to function at work/daily chores, concentration, memory, mood)?

| Not at all | A Little | Somewhat | Much | Very Much |
| --- | --- | --- | --- | --- |
| 0 | 1 | 2 | 3 | 4 |

6. How NOTICEABLE to others do you think your sleeping problem is in terms of impairing the quality of your life?

| Not at all | A Little | Somewhat | Much | Very Much |
| --- | --- | --- | --- | --- |
| 0 | 1 | 2 | 3 | 4 |

7. How WORRIED/distressed are you about your current sleep problem?

| Not at all | A Little | Somewhat | Much | Very Much |
| --- | --- | --- | --- | --- |
| 0 | 1 | 2 | 3 | 4 |
