# Supplementary material for: Migraine patients visiting Chinese medicine hospital: Protocol for a prospective, registry-based, real-world observational cohort study
Source: PLoS One. 2022 Mar 15;17(3):e0265137. doi: 10.1371/journal.pone.0265137 (PMC8923465; doi:10.1371/journal.pone.0265137)
Supplement: S3 File — (DOCX) [file pone.0265137.s004.docx]

**基于真实世界研究的中医治疗偏头痛证据整合**

1. 研究方案摘要：

偏头痛是一种慢性、中-重度原发性头痛，主要临床表现为：反复发作、单侧、搏动性疼痛，常持续4-72小时^[1]^。偏头痛常伴随复杂的并发症，目前尚无根治之法，现代医学对偏头痛的治疗方法效果欠佳，且存在诱发药物过量性偏头痛的风险^[2-6]^。中医以“因人制宜”“辨证论治”为特征^[7]^。针刺和中药是中医最常用的治疗手段，其治疗偏头痛的疗效已被随机临床试验（randomised controlled trials, RCTs）所证实，然而相关证据主要来源于RCT，而RCT常施行严格的纳入排除标准、采用统一的治疗方案，不能反映中医临床实践的实况^[8]^。

面对以上现状，本研究将从电子病历和真实世界队列研究中收集数据，总结中医治疗偏头痛的规律，评估真实临床实践中中医治疗偏头痛的临床效果，同时了解偏头痛患者的医疗需求和关注点。本研究将为基于证据的中医临床实践提供实用性证据支持。

本研究由广东省中医院和澳大利亚皇家墨尔本理工大学联合培养博士项目、中澳国际中医药研究中心支持运行。

1. 立项依据

偏头痛发病率高，兼夹症复杂，现代疗法效果欠佳

偏头痛是一种反复发作的中-重度原发性头痛，主要临床表现为搏动性单侧头痛，常持续4-72小时，且常伴随畏光、畏声、恶心呕吐等症状^[1]^。据报道，其全球发病率高达14.67%^[9]^，且在50岁以下人群的致残率中位列第三^[10]^。偏头痛在澳大利亚的发病率达20.55%，每年造成357亿澳元的经济损失^[9]^。而在中国，偏头痛危害着约1亿5千万人的健康，每年造成五百万伤残损失健康生命年^[11]^。

偏头痛主要分为有先兆的偏头痛和无先兆的偏头痛。先兆症状包括一系列可逆性的视觉、感觉或其它神经系统症状，常发生于头痛之前。偏头痛也可根据发作频率分为发作性偏头痛和慢性偏头痛。若偏头痛发生于经期前后或经期之内，则属于月经相关性偏头痛^[1]^。然而，无论是哪种亚型的偏头痛，目前其病理机制尚未明确。

目前医学尚未有根治偏头痛的方法。其目前治疗方案主要包括急性期对症止痛治疗和预防性治疗，其中，急性期止痛药物包括非特异性止痛药物如阿司匹林、布洛芬等，和偏头痛特异性止痛药如舒马曲坦等；预防性治疗药物包括钙离子拮抗剂、β受体阻滞剂、抗癫痫药等^[12, 13]^。然而，既往调查表明，30-40%的偏头痛患者，特别是慢性偏头痛患者，对现有的西药治疗效果欠满意^[2, 3]^。患者对现有治疗方案不满意之处主要包括药物效力不高、可耐受性低以及药物的不良副反应^[14]^。例如，中国大陆常用的偏头痛预防性药物之一，氟桂利嗪，常导致超重和眩晕^[15]^，而常用的止痛药物非甾体抗炎药则常引起消化道溃疡^[16]^。当偏头痛患者合并失眠、焦虑、抑郁等兼夹症时，患者对药物治疗的满意度则愈加下降^[17-21]^。而且，这些兼夹症的存在，常导致偏头痛治疗药物之间的矛盾，加大治疗难度^[4]^。例如，当偏头痛患者合并抑郁时，其对平素使用的偏头痛药物反应性将下降，且必须避免使用氟桂利嗪等药物，以免加重抑郁^[15, 22]^。除此之外，这些兼夹症的存在，增加了慢性偏头痛的发作率、促进疾病进展^[5, 6]^，而慢性偏头痛患者又常存在过度服用止痛药物的倾向，进而导致药物过量性偏头痛的发生^[1]^。

面对以上种种困局，寻找安全有效的疗法以弥补现行疗法的不足迫在眉睫。

中医治疗偏头痛的有效性证据概况

中医疗法包括中药、针刺、艾灸、穴位按压等，中医治疗头痛、偏头痛在中国历史悠久，可溯源至黄帝内经时代^[23, 24]^，相关知识和经验记载于中医古籍文献中，这些古籍文献所记载的治疗方法可能给现代中医临床实践和临床研究提供较高价值的信息。近年来，中医疗法在全球范围内接受程度日益升高^[25-27]^。据统计，中国大陆约60%的偏头痛门诊患者使用过中医疗法，这一比例与西医疗法不相上下^[28]^。

针刺治疗偏头痛的疗效评价性研究已在世界范围内开展并发表于国际性杂志中。2019年以来发表的相关系统评价提示，针刺是发作性偏头痛的有效治疗手段^[29-34]^。然而月经性偏头痛方面，目前仅有的一篇针刺系统评价，结果提示针刺对月经性偏头痛无效^[35]^。针刺治疗慢性偏头痛方面，目前尚无相关系统性评价，仅一项小型RCT提示针刺对比肉毒杆菌A型注射在治疗慢性偏头痛方面疗效更佳、安全性更高^[36]^。此外，一项回顾性队列研究调查发现，针刺在偏头痛的远期疗效观察中，具有减少医疗费用、减少焦虑抑郁并发症等优势^[37]^。概括而言，目前有充分可信的证据表明针刺治疗发作性偏头痛的有效性和安全性，而针刺治疗慢性偏头痛、月经性偏头痛以及偏头痛并发症等的疗效，有待进一步验证。

中药方面，许多发表于中文期刊的系统评价提示中药在预防性治疗偏头痛和急性止痛方面均有良效^[38-42]^。然而，这些系统评价所纳入的临床研究普遍存在样本量较小、研究方法设计欠严谨、研究质量不高等问题，故而其证据信度不高。仅有一篇基于高质量RCT的系统评价发表于国际期刊，其指出，川芎类方对偏头痛具有良好疗效^[43]^。个别临床研究提示中药对月经性偏头痛有效果^[44-46]^，但中药治疗慢性偏头痛的证据依然匮乏。相比之下，中药治疗偏头痛的实验研究反而得以深入开展，某些中药提取物治疗偏头痛的作用机制得到深入的研究和阐述^[47-49]^。因此，目前仍需要设计完善的高质量临床研究证据促进中药治疗偏头痛的推广。

中医治疗偏头痛的RCT证据外推性、普适性欠佳，而相关真实世界研究证据匮乏

中医基于辨证论治，强调因人制宜^[7]^，体现在临床诊治过程中，可以同病异治，也可以异病同治，辨证为其根本。然而，在西方医学的影响下，验证中医疗效的方法，普遍采用RCT作为检验疗效金标准，而RCT在设计、实施过程中设定严格的纳入排除标准，采用统一的治疗方法^[8]^。由于真实临床实践中的患者病情更为复杂，接受的治疗存在动态性变化，RCT“统一标准”筛选下的研究结果，并不能反映中医“因人制宜”的个性化诊疗效果^[8]^。相比之下，真实世界研究是在真实世界环境下收集与患者有关的数据，与RCT证据大不相同^[50]^。一般认为真实世界证据可以作为RCT证据的补充^[51, 52]^。对于中医研究来说，基于电子病历系统的数据挖掘和在临床实践过程中通过问卷收集的数据，构建临床数据库，将是生成临床假设、为临床决策提供知识支持的有效方法^[53]^。

为了提供更加真实、具有实践指导意义和可信度更高的中医治疗偏头痛的证据，真实世界研究必不可少。

基于证据的临床实践和共同决策成为新的医疗趋势

基于证据的临床实践强调：在有关卫生和社会保健方面的决定需基于现有的、最佳的、有效和相关证据。医疗工作者应向患方提供隐性和显性知识，双方在现有资源范围内共同作出最后决定^[54]^。共同决策是指临床决定需由患者、医护人员根据目前现有最佳证据作出的决定，其不仅需考虑患者所经受的风险和获得利益，还需综合考虑患者的个性和价值观等。共同决策中有三个必不可少的因素：专家经验、医学证据和患者需求及关注点，这是一个将患者的个人目标与医疗证据相结合以获取高质量临床决定的过程^[55]^。

既往研究表明，偏头痛患者最关注的问题包括以下三方面：首先，是疼痛管理相关的生活质量问题，睡眠质量、学习、工作和社交等日常活动受限以及偏头痛给家庭造成的影响；其次，是药物不良反应、药物过量使用和预防性治疗的依赖性；最后，是关于使用预防性药物的相关信息^[13]^。

本研究将通过某些结局指标来反映患者的医疗需求和关注点。因此，本研究将整合专家经验、临床证据和患者需求及关注点，形成中医治疗偏头痛的全方位、立体的证据链，这为基于证据的临床实践和共同决策提供强有力的支持，并在更大程度上造福患者。

1. 研究目的和研究问题

研究目的

本研究的研究目的在于全方位总结现有的中医治疗偏头痛有效性和安全性的证据，整理中医治疗偏头痛的规律。

研究问题

研究问题1

以广东省中医院为例，在真实中医临床实践中，中医治疗偏头痛有什么模式和规律特征？

研究问题2

以广东省中医院为例，在真实中医临床实践中，中医治疗偏头痛的临床疗效如何？有哪些影响因素？患者有何医疗需求和关注点？

1. 研究方法

为解答以上问题，本研究将开展以下两个项目。

第一部分:中医治疗偏头痛的规律和处方特点分析：基于电子病历系统的回顾性研究

研究目标

本研究主要基于广东省中医院^[56]^电子病历系统，对偏头痛的诊治规律、处方特点等进行回顾性研究，同时也对偏头痛患者治疗方式的选择规律进行探索和总结。

研究设计

本研究将基于2018年7月-2020年7月期间，广东省中医院门诊病历系统所记载的第一西医诊断为偏头痛的病历进行回顾性分析。本研究为基于既往偏头痛病历中的病史和诊疗信息的回顾性分析。研究者没有权限接触患者的姓名、身份证、家庭地址、出生日期、联系电话等敏感性信息，无法对每一份病历相关的患者进行告知及知情同意的获取，且本研究不涉及患者的私密信息，对患者无可预见性危害或风险，故而申请免除知情同意。

纳入排除标准

#### 纳入标准

- 偏头痛为第一诊断。
- 包含有详细治疗信息。

#### 排除标准

- 继发性偏头痛，病历描述提示头痛由头部外伤、脑部肿瘤、青光眼、外感等疾病继发。
- 孕妇。
- 以开药为目的，且所开药物与偏头痛无关的病历记录。
- 就诊主诉非头痛的病历记录。
- 头痛症状描述明显不符合ICHD-3中偏头痛诊断标准。

数据收集和准备

#### 数据导出

原始电子病历将由广东省中医院信息处以RTF文档的形式从病历系统中导出。每一位患者的每一次就诊记录为一份独立的文档。

#### 病历筛选

首先，通过阅读诊断、治疗信息，根据纳入和排除标准筛选合格病历。

#### 分类提取信息

患者的诊疗卡号、性别、年龄、就诊日期、籍贯、就诊科室、处方医生的职称、主诉、现病史、既往史、过敏史、家族史、月经史、中医诊断、中医证型、西医诊断、中西医治疗方案（药物、剂量、频次及疗程）等信息将被提取到Excel文档中，同一个患者的不同诊次将按时间先后顺序排列。

#### 进一步信息分解

患者的伴随症状如睡眠障碍、焦虑、抑郁等，其它偏头痛特征性临床表现如恶心呕吐、畏光畏声等，以及偏头痛病程、疼痛程度、发作频率、首发年龄、止痛药使用情况、治疗效果等详细的信息都将被进一步提取。另外，患者的就诊频次、疗程也将被记录分析。此外，病程记录中有关病情改善、头痛减轻等提示临床症状改善的词汇将进一步提取标志，以供进一步分析。

治疗处方中的中成药当独立分列，说明书中的药物组成也将详细记录。类似的，中药汤剂中的每一味中药、针灸处方中的每一个穴位都详细记录。

#### 标准化处理

在数据的转换过程中，若一种中药同时有多个名称时，根据《中药学》^[57]^和《中国药典》^[58]^进行规范化命名。若是同一种中药经过不同方法炮制而产生不同的功效，则记录原名称。

隐私保护

以上提取的电子数据将存储于设密的澳大利亚皇家墨尔本理工大学提供的电脑云盘中，至少存储至研究结果公布后5年；同时将永久性储存于广东省中医院网盘中。以上数据仅课题组成员有权限查阅，并仅限课题组成员使用。

以上研究过程均由经伦理委员会审查通过的研究人员负责。

数据分析

使用IBM SPSS26.0^[59]^统计软件分析。定量资料如年龄、偏头痛发作频次、病程、疗程等，进行正态分布检验，若数据呈正态分布，将以均数和标准差来描述数据特征，并运用t检验或方差分析；非正态分布数据则以中位数、四分位差来描述数据特征，运用秩和检验进行组间差异性检验。定性资料如偏头痛类型、治疗方式等，则以频数和百分比来描述数据，运用卡方检验或F检验进行组间比较。采用双侧检验法，选取P<0.05为显著性差异的标准。

此外，治疗药物及穴位将进行频次分析、聚类分析、相关规则分析以展现中医治疗的规律。另外，还将探索性运用回归分析方法来探索影响治疗方式选择和疗程长短的因素、影响偏头痛临床表现的人口学特征因素。最后，有记录临床症状改善的病历将筛选出来以分析“临床有效医案”的诊治规律。

研究伦理问题的考虑

本部分研究基于既往电子病历分析偏头痛中医诊疗规律，不涉及患者个人姓名、出生年月、身份证号码、详细地址和联系方式等私密、敏感信息，在数据处理及分析、呈现部分不涉及患者的身份信息，对患者的健康、名誉、心理、经济等不造成伤害或风险。

第二部分：基于真实世界的中药治疗偏头痛的前瞻性登记注册队列观察

研究目标

本部分研究将基于真实世界，通过前瞻性随访观察首次因偏头痛就诊于广东省中医院头痛门诊的偏头痛患者，总结中医治疗偏头痛的规律。其次，本研究将探讨中医治疗暴露程度与临床效果，特别是止痛药使用情况之间的相关性。再者，本研究将探索与中医治疗偏头痛临床疗效密切相关的潜在因素。同时，本部分研究也通过生活质量评价等结局指标反映患者的医疗需求和关注点。概括而言，本部分研究将涉及基于证据的临床实践三个必要因素：专家意见、临床证据和患者医疗需求及关注点，分别通过诊疗规律总结、良好的队列观察设计、结合患者个人感受的结局指标等进行呈现。基于广东省中医院官方公布的诊疗记录信息，中药为大部分偏头痛患者选择的治疗方式，故中药为本部分研究主要观察目标。

研究设计

本登记注册研究将于广东省中医院头痛门诊^[56]^进行，研究时间为2020年12月-2022年5月，为期1年半。符合纳入排除标准的受试者将被连续性纳入。招募广告将被张贴于头痛门诊候诊厅内，简单介绍本研究的纳入标准及研究者的联系方式。当潜在受试者首次就诊时，主诊医师向患者进行偏头痛的宣教以及规律监测病情的重要性，建议患者可参与本研究。研究者向潜在受试者进一步介绍研究计划，告知其需承担一定的时间成本配合进行为期12周的随访，每4周进行一次评价。在受试者自主意愿之下签署知情同意书后，进行本观察研究。若观察对象为研究者诊治对象时，则知情同意过程及数据收集过程由研究助理完成，同时知情同意过程中，需第三方在场并签字同意。首次就诊时，受试者需填写基本信息调查表。偏头痛相关调查表则于入组时、每次随访均填写。数据将在组间进行比较，而分组标准可灵活变化，可根据疗程分组，治疗方式分组或其他临床特征进行分组。具体研究过程详见图1。


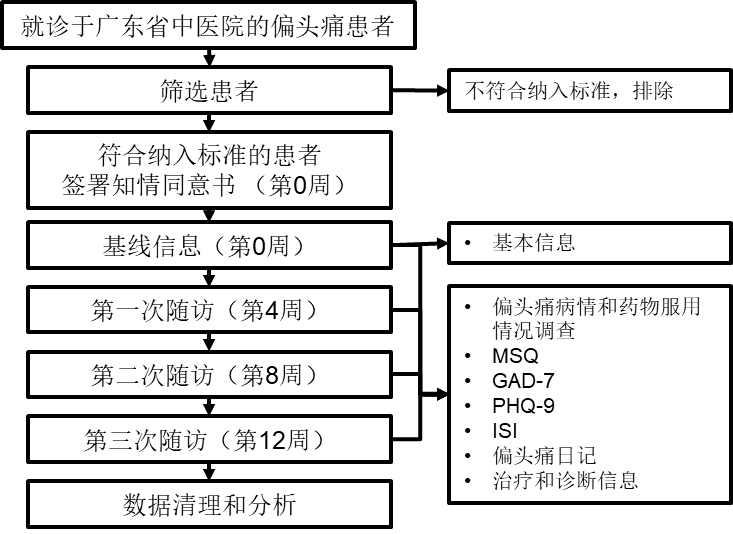


图1：队列研究流程图

注：GAD-7: Generalised Anxiety Disorder 7-item Scale，广泛性焦虑量表；GPHCM: Guangdong Provincial Hospital of Chinese Medicine，广东省中医院；ISI: Insomnia Severity Index Scale，失眠指数；MSQ: Migraine Specific Questionnaire，偏头痛生活质量评价；PHQ-9: Patient Health Questionnaire-9，患者健康问卷。

纳入排除标准

#### 纳入标准

1. 偏头痛为第一诊断，且符合国际头痛协会制定的头痛分类标准-3^[1]^。
2. 首次因偏头痛至广东省中医院寻求治疗的患者。
3. 签署知情同意书。
4. 年龄大于18周岁。
5. 未同时参与其他干预性临床研究。

#### 排除标准

1. 既往诊断过青光眼、脑部肿瘤、头部外伤等可能引起偏头痛的疾病。
2. 患者具有严重的视力、听力、语言、智力、记忆、意识障碍等，无法配合完成调查表的填写与随访。
3. 孕妇。
4. 高度依赖医疗护理。

#### 终止观察标准

1. 由于主观或客观原因失去观察对象的联系。
2. 完成12周随访观察。
3. 诊疗过程中修正偏头痛诊断。

基本信息

患者的人口学信息和基本背景信息将在入组时根据患者的个人偏好，通过面对面问卷调查或线上问卷调查进行收集。相关资料若有缺项，研究者将在当天或隔天检查发现后联系患者进行补充。无论是通过何种方式收集的数据，最后都将输入到Excel表中。需要收集的基本信息详见附件中问卷1。

观察指标

#### 观察指标

根据偏头痛临床研究指南^[60]^和研究目的，本研究的观察指标详见如下：

偏头痛发作频率（次/4周），有效率（发作频率减少一半或以上为有效），头痛天数（天/4周），头痛持续时间（平均每次头痛持续时间），头痛程度NRS分数，止痛药剂量（运用药物量化表，medication quantification scale^[61]^），偏头痛生活质量量表（migraine specific questionnaire，MSQ）^[62]^，广泛性焦虑障碍量表（Generalized Anxiety Disorder 7-item scale，GAD-7）^[63]^，患者健康问卷（Patient Health Questionnaire-9，PHQ-9）^[64]^，失眠指数（insomnia severity index Scale，ISI）^[65, 66]^，患者就诊关注点，疗效评价的5点李克特量表。观察过程中，若患者PHQ-9得分属于高风险范畴，则及时向该患者的主诊医生反馈，并建议患者心理专科随诊。

不良反应：治疗过程中，患者出现的任何可疑不良反应，如腹泻、口腔溃疡、失眠等，均建议记录于头痛日记中，最后汇总分析。

#### 治疗和诊断信息

除了以上由患者填写的问卷之外，患者的详细治疗信息和既往诊断信息等内容，将由研究者通过查询病历进行记录。若随访观察期间患者另外就医，可依据其病历记录资料进行收集（详见附录1）。此外，若观察对象因客观原因不能独立完成问卷量表填写，可由研究者通过电话或面对面辅助解释并获取相关数据。

数据收集主要通过问卷调查的形式进行。具体问卷观察点安排详见表1。

表1：问卷及观察点安排

| 时间 | 基本信息 | 头痛病情及药物使用情况 | MSQ | GAD-7 | PHQ-9 | ISI | 诊断和治疗信息 | 头痛日记 |
| --- | --- | --- | --- | --- | --- | --- | --- | --- |
| 基线（第0周） | √ | √ | √ | √ | √ | √ | √ | √ |
| 第1次随访（第4周） |  | √ | √ | √ | √ | √ | √ | √ |
| 第2次随访（第8周） |  | √ | √ | √ | √ | √ | √ | √ |
| 第3次随访（第12周） |  | √ | √ | √ | √ | √ | √ | √ |

注：GAD: Generalized Anxiety Disorder 7-item Scale，广泛性焦虑障碍量表；ISI：Insomnia Severity Index Scale，失眠严重指数；MSQ：Migraine Specific Quality of Life，偏头痛生活质量评价；PHQ-9：Patient Health Questionnaire-9，患者健康问卷。

样本量及纳入方法

由于本研究是基于真实世界的登记注册队列观察，主要目的在于基于广东省中医院偏头痛患者人群信息探索中药治疗偏头痛的规律和疗效，没有特定的研究假说需要验证。基于医院官方既往公布的门诊就诊记录显示，结合本研究目的，预计本研究大约纳入400人。本研究的观察对象将以连续纳入的方式进行登记注册，以减少选择误差^[67]^。

队列分析中可能运用的暴露因素

本队列研究中，将根据治疗方式、疗程、偏头痛亚型（发作性偏头痛vs慢性偏头痛，月经性偏头痛vs非月经性偏头痛）、偏头痛伴发症情况（根据GAD-7、PHQ-9和ISI的分数分层）、基线特征（病程、基线头痛频率、基线头痛程度和止痛药使用情况）等因素进行分组或分层。

隐私保护

本研究产生的相关数据将存储于设密的澳大利亚皇家墨尔本理工大学提供的电脑云盘中，至少存储至研究结果公布后5年；同时将永久性储存于广东省中医院网盘中。以上数据仅课题组成员有权限查阅，并仅限课题组成员使用。在研究过程中若发生严重不良事件，广东省中医院伦理委员可查阅相关研究数据。

以上研究过程均由经伦理委员会审查通过的研究人员负责。

统计分析

#### 分析方法

定性资料如性别、偏头痛类型等，将通过频次和百分比进行描述，组间比较运用卡方检验或Fisher检验；定量资料如病程、头痛频率、头痛程度等，若符合正态分布，则通过均数和标准差进行描述，否则通过中位数和四分位数差进行描述数据分布特征，前者运用t检验或多因素方差分析进行组间比较，后者运用秩和检验进行组间比较。所有组间差异比较为双侧检验，P<0.05为显著性差异标准。

此外，本研究将运用相关分析与多元回归分析探索与临床效果相关的预测因素。重复测量方差分析（Repeated measure ANOVA）则被运用来检验组内不同在不同观察时间的结局指标之间的差异性，若某一结局指标为分类变量或不符合方差分析的连续性变量，则采用广义估计方程（generalised estimated equation，GEE）进行检验。聚类分析和关联规则分析等其他分析方法也可能运用于数据探索分析过程中。

以上统计分析将运用IBM SPSS 26.0统计软件进行。

#### 潜在偏倚及其控制方法

首先，在观察对象招募阶段，采用连续性纳入方法以减少选择偏倚，同时，根据指南的诊断标准评估病人诊断以减少误诊偏倚。

在观察随访阶段，制定、指导患者适用头痛日记，以提高数据真实性，减少随访时患者出现回忆误差。

由于本研究的随访时长达12周，数据缺失难以避免。本研究将采用多元化的随访手段，包括面对面随访、网络随访、电话随访等，以减少失访率。另外，在每个随访点到来之时，研究者将通过如微信、电话、短信等方式及时提醒患者，减少失访率。对于每份问卷，在回收之时，及时检查有无缺漏的项目，及时通过电话等方式向观察对象询问、补充相关信息。

研究伦理问题的考虑

本部分研究基于中医临床实践过程观察患者特征、治疗方案及疗效进行观察和评价，对患者没有附加的治疗、检查，没有给患者造成额外的经济负担或与治疗相关的风险。在数据收集、分析、处理阶段，对患者的身份信息进行重新编码，保护患者的隐私。数据收集不涉及患者的敏感性信息。患者的联系方式将储存于密码保护的电子设备中，仅本课题组成员基于研究目的方可联系患者。本研究对患者的影响，在于患者需要额外的时间配合完成问卷的填写和头痛日记的记录，健康和诊疗信息被利用分析，联系方式被记录并运用于研究过程。但考虑通过本研究，患者对偏头痛这一疾病的认识将得到加深，并获得对偏头痛病情监测和自我管理的能力，研究结果将可能造福广大偏头痛患者，具有一定的相关个人、社会利益。综合考虑，本研究的利大于弊。

1. 预期成果

- 基于广东省中医院既往偏头痛的电子病历，总结中医疗法治疗偏头痛的真实临床实践规律。
- 基于前瞻性队列研究的结果，总结中药治疗偏头痛的疗效和安全性；同时还将总结偏头痛患者在寻求中医治疗时的个人需求、目标和关注点，为临床决策提供证据。

1. 研究意义

偏头痛是一种发病率高的慢性致残性疾病，由于其临床表现的复杂性、伴随疾病的多样性以及药物过量性偏头痛等风险，目前药物治疗难度大，疗效不满意。中医是强调“辨证论治”“因人制宜”的医学体系，同时强调“整体观”，将同时患多种疾病、出现多种症状的患者看成一个整体进行治疗。既往研究表明，以针刺和中药为代表的中医疗法治疗典型偏头痛具有一定的效果，然而既往证据主要来源于RCT，而RCT在实施过程中常制定严格的纳排标准和统一的治疗方案，而偏头痛患者病情常较复杂，这种研究方式下得出证据在临床实践中实用性欠佳。

在基于证据的临床实践和共同决策理念指导下，结合前期的古籍文献和现代文献系统评价工作，本研究通过古籍文献和真实世界病历的数据挖掘总结专家经验，通过系统评价和队列研究整合临床研究证据，通过患者报道的结局指标整合偏头痛患者的医疗需求和关注点，从而提供全方位、立体的证据力，为中医治疗偏头痛的临床决策提供有力的证据支持，更大程度上造福广发偏头痛患者。

1. 研究进程安排

**表2：研究进程安排**

| 项目 | 预计时间 | 项目 | 预计时间 |
| --- | --- | --- | --- |
| 前瞻性队列研究 | | 回顾性病历分析 | |
| 伦理申请 | 2020.10.25-2020.11.30 | 伦理申请 | 2020.10.25-2020.11.30 |
| 招募受试者 | 2020.12.01-2022.02.28 | 导出病历资料 | 2020.12.01-2020.12.15 |
| 随访观察 | 2020.12.01-2022.05.31 | 数据整理 | 2020.12.16-2021.02.28 |
| 数据整理 | 2022.03.01-2022.06.30 | 研究报告撰写 | 2021.03.01-2021.06.30 |
| 研究报告撰写 | 2022.07.01-2022.09.30 | 论文发表 | 2021.07.01-2021.12.30 |
| 论文发表 | 2022.10.01-2022.12.30 |  |  |

1. 课题组成员及分工

| 姓名 | 单位 | 学历 | 职责 | 与该项目是否存在利益冲突 |
| --- | --- | --- | --- | --- |
| 吕少华 | 广东省中医院，澳大利亚皇家墨尔本理工大学 | 硕士 | 设计、实施本研究，对受试者进行招募、观察、随访，收集数据，数据统计分析、研究报告撰写 | 否 |
| 郭新峰 | 广东省中医院 | 博士 | 指导研究方案设计，质量控制 | 否 |
| 孙景波 | 广东省中医院 | 博士 | 临床观察沟通协调，质量控制 | 否 |
| Charlie Changli Xue | 澳大利亚皇家墨尔本理工大学 | 博士 | 指导研究方案设计 | 否 |
| Anthony Lin Zhang | 澳大利亚皇家墨尔本理工大学 | 博士 | 指导研究方案设计和执行，指导数据管理和分析 | 否 |
| Claire Shuiqing Zhang | 澳大利亚皇家墨尔本理工大学 | 博士 | 指导研究方案设计和执行，指导数据管理和分析 | 否 |
| 毛振辉 | 广东省中医院 | 学士 | 研究助理，辅助进行受试者招募、观察、随访、收集数据 | 否 |

1. 附录

问卷1：基本信息调查表

问卷2：偏头痛病情及药物使用情况调查表

问卷3：偏头痛生活质量量表

问卷4：广泛焦虑障碍量表

问卷5：病人健康抑郁障碍量表

问卷6：失眠严重指数

偏头痛日记

诊断及治疗信息表

随访阶段偏头痛病情及药物使用情况调查表

1. 参考文献：

[1] HEADACHE CLASSIFICATION COMMITTEE OF THE INTERNATIONAL HEADACHE SOCIETY (IHS). The International Classification of Headache Disorders 3rd edition [J]. Cephalalgia, 2018, Jan;38(1):1-211.(

[2] LIPTON R B, BUSE D C, SERRANO D, et al. Examination of unmet treatment needs among persons with episodic migraine: results of the American Migraine Prevalence and Prevention (AMPP) Study [J]. Headache, 2013, 53(8): 1300-11.

[3] YOUNG N P, PHILPOT L M, VIERKANT R A, et al. Episodic and Chronic Migraine in Primary Care [J]. Headache, 2019, 59(7): 1042-51.

[4] FINOCCHI C, VILLANI V, CASUCCI G. Therapeutic strategies in migraine patients with mood and anxiety disorders: clinical evidence [J]. Neurol Sci, 2010, 31 Suppl 1(S95-8.

[5] DIENER H C, HOLLE D, SOLBACH K, et al. Medication-overuse headache: risk factors, pathophysiology and management [J]. Nature reviews Neurology, 2016, 12(10): 575-83.

[6] MINEN M T, BEGASSE DE DHAEM O, KROON VAN DIEST A, et al. Migraine and its psychiatric comorbidities [J]. Journal of neurology, neurosurgery, and psychiatry, 2016, 87(7): 741-9.

[7] CAO H. Basic theory of traditional Chinese medicine [M]. Beijing: China Press of Traditional Chinese Medicine, 2004.

[8] BLACK N. Why we need observational studies to evaluate the effectiveness of health care [J]. BMJ (Clinical research ed), 1996, 312(7040): 1215-8.

[9] DELOITTE ACCESS ECONOMICS. Migraine in Australia Whitepaper [M]. 2018.

[10] STEINER T J, STOVNER L J, VOS T. GBD 2015: migraine is the third cause of disability in under 50s [J]. J Headache Pain, 2016, 17(1): 104.

[11] YAO C, WANG Y, WANG L, et al. Burden of headache disorders in China, 1990-2017: findings from the Global Burden of Disease Study 2017 [J]. J Headache Pain, 2019, 20(1): 102.

[12] CEPHALALGIA GROUP PAIN-RELIEVING BRANCH CHINESE MEDICAL ASSOCIATION. Guideline for migraine in China [J]. Chinese Journal of Pain Medicine, 2016, 22(10):

[13] SIGN 155 • Pharmacological management of migraine [M]. 2018.

[14] MARCI CLARK T J S, STEWART J TEPPER, NIMANEE HARRIS,SUSAN MARTIN, SANDHYA SAPRA , NEEL SHAH. Patient satisfaction with prophylactic migraine medications [M]. the 59th Annual Scientific Meeting American Headache Society. Boston, MA. 2017.

[15] PEER MOHAMED B, GOADSBY P J, PRABHAKAR P. Safety and efficacy of flunarizine in childhood migraine: 11 years' experience, with emphasis on its effect in hemiplegic migraine [J]. Developmental medicine and child neurology, 2012, 54(3): 274-7.

[16] PARDUTZ A, SCHOENEN J. NSAIDs in the Acute Treatment of Migraine: A Review of Clinical and Experimental Data [J]. Pharmaceuticals (Basel), 2010, 3(6): 1966-87.

[17] TORTA R, IERACI V. Migraine and depression comorbidity: antidepressant options [J]. Neurol Sci, 2012, 33 Suppl 1(S117-8.

[18] VETVIK K G, MACGREGOR E A. Sex differences in the epidemiology, clinical features, and pathophysiology of migraine [J]. The Lancet Neurology, 2017, 16(1): 76-87.

[19] KOZAK H H, BOYSAN M, UCA A U, et al. Sleep quality, morningness-eveningness preference, mood profile, and levels of serum melatonin in migraine patients_ a case-control study [J]. Acta neurologica Belgica, 2017, 117(1): 111-9.

[20] ALSTADHAUG K S R, BEKKELUND S. Insomnia and circadian variation of attacks in episodic migraine [J]. Headache, 2007, 47(8): 1184-8.

[21] JIYOUNG KIM S-J C, WON-JOO KIM, KWANG IK YANG, CHANG-HO YUN AND MIN KYUNG CHU. Insufficient sleep is prevalent among migraineurs: a population-based study [J]. The Journal of Headache and Pain, 2017, 18(1): 50.

[22] VERSPEELT J, DE LOCHT P, AMERY W K. Post-marketing cohort study comparing the safety and efficacy of flunarizine and propranolol in the prophylaxis of migraine [J]. Cephalalgia : an international journal of headache, 1996, 16(5): 328-36; discussion 288.

[23] ANJI LIN Y W. Chinese medicine for 'Toufeng': a literature review; proceedings of the The first International Forum on the Development of Traditional Chinese Medicine, the First International Symposium on the Prevention and Treatment of AIDS by Traditional Chinese Medicine, and the Association of Directors of National Scientific Research Institutes of Traditional Chinese Medicine, Beijing China, F, 2005 [C].

[24] WANG W. The regularition of Chinese medicine for migraine: report based on medical records [D]; Guangzhou University of Traditional Chinese Medicine, 2017.

[25] ITALIA S, BRAND H, HEINRICH J, et al. Utilization of complementary and alternative medicine (CAM) among children from a German birth cohort (GINIplus): patterns, costs, and trends of use [J]. BMC complementary and alternative medicine, 2015, 15(49.

[26] YANG L, ADAMS J, SIBBRITT D. Prevalence and factors associated with the use of acupuncture and Chinese medicine: results of a nationally representative survey of 17161 Australian women [J]. Acupuncture in medicine : journal of the British Medical Acupuncture Society, 2017, 35(3): 189-99.

[27] DE MORAES MELLO BOCCOLINI P, SIQUEIRA BOCCOLINI C. Prevalence of complementary and alternative medicine (CAM) use in Brazil [J]. BMC Complement Med Ther, 2020, 20(1): 51.

[28] YU S, ZHANG Y, YAO Y, et al. Migraine treatment and healthcare costs: retrospective analysis of the China Health Insurance Research Association (CHIRA) database [J]. J Headache Pain, 2020, 21(1): 53.

[29] ZHANG N, HOULE T, HINDIYEH N, et al. Systematic Review: Acupuncture vs Standard Pharmacological Therapy for Migraine Prevention [J]. Headache, 2020, 60(2): 309-17.

[30] CHEN Y Y, LI J, CHEN M, et al. Acupuncture versus propranolol in migraine prophylaxis: an indirect treatment comparison meta-analysis [J]. Journal of neurology, 2020, 267(1): 14-25.

[31] LI Y X, XIAO X L, ZHONG D L, et al. Effectiveness and Safety of Acupuncture for Migraine: An Overview of Systematic Reviews [J]. Pain research & management, 2020, 2020(3825617.

[32] TRINH K V, DIEP D, CHEN K J Q. Systematic Review of Episodic Migraine Prophylaxis: Efficacy of Conventional Treatments Used in Comparisons with Acupuncture [J]. Medical acupuncture, 2019, 31(2): 85-97.

[33] LI X, DAI Q, SHI Z, et al. Clinical Efficacy and Safety of Electroacupuncture in Migraine Treatment: A Systematic Review and Network Meta-Analysis [J]. The American journal of Chinese medicine, 2019, 47(8): 1755-80.

[34] ZHANG X T, LI X Y, ZHAO C, et al. An Overview of Systematic Reviews of Randomized Controlled Trials on Acupuncture Treating Migraine [J]. Pain research & management, 2019, 2019(5930627.

[35] YANG M, DU T, LONG H, et al. Acupuncture for menstrual migraine: a systematic review [J]. BMJ supportive & palliative care, 2020,

[36] NADERINABI B, SABERI A, HASHEMI M, et al. Acupuncture and botulinum toxin A injection in the treatment of chronic migraine: A randomized controlled study [J]. Caspian journal of internal medicine, 2017, 8(3): 196-204.

[37] LIAO C C, LIAO K R, LIN C L, et al. Long-Term Effect of Acupuncture on the Medical Expenditure and Risk of Depression and Anxiety in Migraine Patients: A Retrospective Cohort Study [J]. Frontiers in neurology, 2020, 11(321.

[38] JUENING YAO B Z, KEGANG CAO. Effect of external application of Chinese medicine on acupoint for migraine: a systematic review based on RCTs [J]. World Traditional Chinese Medicine, 2019, 14(11): 2930-4.

[39] WEIDONG LUO J W, YAXIAN CAI, GUOHUA CHEN. Effectiveness and safert of intergrated Chinese medicine and western medicine for migraine: a systematic review and meta-analysis [J]. Hunan Journal of Traditional Chinese Medicine, 2018, 34(07): 157-60.

[40] YINHE CAI K L, WEIPENG SUN, ZHIBING WU. Effectiveness of Tianmagouteng formula compared with CCB for migraine: a systematic review and meta-analysis [J]. Chinese Journal of Basic Medicine of TCM, 2018, 24(07): 949-54.

[41] MENGUO YUAN Y L, TING TIAN, WEIFENG GUO. Effectiveness of the method of activating blood and removing wind for migraine: a systematic review and meta-analysis [J]. Guiding Journal of Tradication Chinese Medicine, 2017, 23(01): 73-5+9.

[42] XIAOWEN YU G L, ZHONGLIN WANG. Effectiveness of Tongqiaohuoxue formular for migraine: a systematic review based on RCTs [J]. Journal of Shandong University of Chinese Medicine, 2017, 41(03): 202-6.

[43] SHAN C S, XU Q Q, SHI Y H, et al. Chuanxiong Formulae for Migraine: A Systematic Review and Meta-Analysis of High-Quality Randomized Controlled Trials [J]. Frontiers in pharmacology, 2018, 9(589.

[44] SHOURAN LI W N, JIN WEN. Effect of Chaihuguizhi formular for menstrual migraine: a clinical trial [J]. Jilin Journal of Tradication Chinese Medicine, 2018, 38(9):

[45] DUAN Z. Case series of Tongqiaohuoxue Formula for menstrual migraine [J]. Henan Journal of Chinese medicine, 2013, 33(11):

[46] SHUN XIU WU C Y X, XIAN GUANG CHEN, SEN MEI LI, LI LING WEI. Injection of Yimucao for menstrual migraine: a clinical trial [J]. Liaoning Journal of Traditional Chinese Medicine, 2004, 31(12):

[47] LAI T, CHEN L, CHEN X, et al. Rhynchophylline attenuates migraine in trigeminal nucleus caudalis in nitroglycerin-induced rat model by inhibiting MAPK/NF-кB signaling [J]. Mol Cell Biochem, 2019, 461(1-2): 205-12.

[48] LIU Z K, NG C F, SHIU H T, et al. Neuroprotective effect of Da Chuanxiong Formula against cognitive and motor deficits in a rat controlled cortical impact model of traumatic brain injury [J]. Journal of ethnopharmacology, 2018, 217(11-22.

[49] GUAN J, ZHANG X, FENG B, et al. Simultaneous determination of ferulic acid and gastrodin of Tianshu Capsule in rat plasma by ultra-fast liquid chromatography with tandem mass spectrometry and its application to a comparative pharmacokinetic study in normal and migraine rats [J]. J Sep Sci, 2017, 40(21): 4120-7.

[50] GARRISON JR L P, NEUMANN P J, ERICKSON P, et al. Using real‐world data for coverage and payment decisions: The ISPOR real‐world data task force report [J]. Value in health, 2007, 10(5): 326-35.

[51] SHERMAN R E, ANDERSON S A, DAL PAN G J, et al. Real-World Evidence - What Is It and What Can It Tell Us? [J]. N Engl J Med, 2016, 375(23): 2293-7.

[52] GROOTENDORST D C, JAGER K J, ZOCCALI C, et al. Observational studies are complementary to randomized controlled trials [J]. Nephron Clin Pract, 2010, 114(3): c173-7.

[53] LIU B, ZHOU X, WANG Y, et al. Data processing and analysis in real-world traditional Chinese medicine clinical data: challenges and approaches [J]. Stat Med, 2012, 31(7): 653-60.

[54] DAWES M, SUMMERSKILL W, GLASZIOU P, et al. Sicily statement on evidence-based practice [J]. BMC Med Educ, 2005, 5(1): 1.

[55] ALSTON C, PAGET L, HALVORSON G, et al. Communicating with patients on health care evidence [J]. NAM Perspectives, 2012,

[56] 广东省中医院[M]. 广州中医大学网络有限公司. http://www.gdhtcm.com/index.html.

[57] 高学敏. 中药学[M]. 中国中医药出版社, 2000.

[58] STATE PHARMACOPOEIA COMMITTEE OF CHINA. Chinese Pharmacopoeia [M]. 2015.

[59] IBM CORP. IBM SPSS Statistics for Windows [M]. Armonk, NY; IBM Corp. 2017.

[60] TFELT-HANSEN P, PASCUAL J, RAMADAN N, et al. Guidelines for controlled trials of drugs in migraine: third edition. A guide for investigators [J]. Cephalalgia, 2012, 32(1): 6-38.

[61] HARDEN R N, WEINLAND S R, REMBLE T A, et al. Medication Quantification Scale Version III: update in medication classes and revised detriment weights by survey of American Pain Society Physicians [J]. The journal of pain : official journal of the American Pain Society, 2005, 6(6): 364-71.

[62] MARTIN B C, PATHAK D S, SHARFMAN M I, et al. Validity and reliability of the migraine‐specific quality of life questionnaire (MSQ Version 2.1) [J]. Headache: The Journal of Head and Face Pain, 2000, 40(3): 204-16.

[63] SPITZER R L, KROENKE K, WILLIAMS J B W, et al. A Brief Measure for Assessing Generalized Anxiety Disorder: The GAD-7 [J]. Archives of Internal Medicine, 2006, 166(10): 1092-7.

[64] KROENKE K, SPITZER R L, WILLIAMS J B. The PHQ-9: validity of a brief depression severity measure [J]. J Gen Intern Med, 2001, 16(9): 606-13.

[65] MORIN C M, BELLEVILLE G, BÉLANGER L, et al. The Insomnia Severity Index: Psychometric Indicators to Detect Insomnia Cases and Evaluate Treatment Response [J]. Sleep, 2011, 34(5): 601-8.

[66] BAGHYAHI S B A, GAO Y, TAGHANAKI H B, et al. 2738–Reliability and validity of the chinese translation of insomnia severity index (C-ISI) in chinese patients with insomnia [J]. European Psychiatry, 2013, 28(S1): 1-.

[67] GLIKLICH RE D N, LEAVY MB. Registries for Evaluating Patient Outcomes: A User's Guide [M/OL]. April 2014[<https://www.ncbi.nlm.nih.gov/books/NBK208632/>.
