# Supplementary material for: Migraine patients visiting Chinese medicine hospital: Protocol for a prospective, registry-based, real-world observational cohort study
Source: PLoS One. 2022 Mar 15;17(3):e0265137. doi: 10.1371/journal.pone.0265137 (PMC8923465; doi:10.1371/journal.pone.0265137)
Supplement: S4 File — (DOCX) [file pone.0265137.s005.docx]

**Chinese medicine for migraine: a prospective registry-based cohort study based on real-world clinical practice and a retrospective study based on medical records**

1. Abstract

Migraine is a chronic, severe and primary headache disorder, manifesting in recurrent, unilateral, throbbing headache lasting for 4 to 72 hours [1]. Migraine is accompanied with complex comorbidities and currently there is no cure for this disease. The effects of pharmacotherapy for migraine are unsatisfying, and it is associated with potential risk of medication overuse [2-6]. Chinese medicine (CM), features in individualised treatment regime based on each patent’s specific symptoms [7]. Acupuncture and Chinese herbal medicine (CHM), have been evidenced to be effective and safe in treating typical migraine based on data generated from randomised controlled trials (RCTs). However, the approach of applying strict eligible criteria and unified treatment in RCTs does not reflect the features of CM in real-world clinical practice [8].

This project is designed to summarise real-world evidence of effectiveness and safety of CM for migraine. It consists two components: retrospectively analysing data of medical records to reveal the real utilisations of CM for migraine, and prospectively registering migraineurs in a cohort study to generate real-world evidence of CHM for migraine. Also, the project will summarise the concerns and preferences of patients towards CM. The project will provide practical evidence to support evidence-based Chinese medicine practice for migraine.

The study is supported Guangdong Provincial Hospital of Chinese Medicine (GPHCM) and China-Australia Research Centre of Chinese Medicine.

1. Rationale

High prevalence, challenging complexity, and unsatisfying treatment effect

Migraine is a recurrent, severe and primary headache disorder, manifesting in unilateral, throbbing headache lasting for 4 to 72 hours. And it is often accompanied with phonophobia, photophobia, and vomiting, etc [1]. Migraine has been reported to present a global prevalence of 14.67% [9] and ranked as the third cause of disability among population under 50’s worldwide [10]. As reported in 2018, migraine had a prevalence of 20.55% and induced $35.7 billion economic loss in Australia [9]. In China, migraine was estimated to affect almost 150 million people and caused 5.5 million years lived with disability in 2017 [11].

Migraine is categorised as migraine with aura and migraine without aura. Aura is the reversible visual, sensory or other central nervous system symptoms preceding headache. Migraine is also classified as episodic migraine and chronic migraine, depending on the migraine attacks frequency. Another specific type is menstrual migraine, which mainly attacks around menstrual cycle [1]. Regardless the subtypes, the pathophysiological mechanisms of migraine remain unclear.

Currently there is no cure for migraine. Migraine is usually managed by medications for relieving symptoms in the acute stage, and by preventive treatment for reducing the frequency and severity of migraine attacks. The commonly used medications are nonsteroidal anti-inflammatory drugs (NSAIDs), triptans, calcium channel blocker like flunarizine, β receiver inhibitor like propranolol, etc [12, 13]. However, approximately 30-40% migraineurs are not satisfied with the current pharmacotherapies, especially those suffer from chronic migraine [2, 3], due to the insufficient treatment effects, low tolerability and side effects of the medications [14]. For example, overweight and dizziness are commonly reported adverse effects caused by flunarizine [15], and gastrointestinal ulcer is associated with NSAIDs [16]. In particular, among those comorbid with anxiety, depression and insomnia, the degree of dissatisfaction of migraine medications is higher than those without comorbidities [17-21]. These comorbidities make it more difficult in selecting medications for migraine management [4]. For instance, migraineurs comorbid with depression often do not respond to routine management, and also are suggested to avoid flunarizine due to its potential risk of exacerbating depression [15, 22]. Furthermore, such comorbidities increase the risks of the occurrence and progress of chronic migraine [5, 6], which may further result in medication overuse headache [1].

Acknowledging challenges above, there is an urgent need of identifying other effective and safe therapies to assist the current migraine management.

Current evidence of Chinese medicine therapies for migraine

CM therapies consist of CHM, acupuncture, moxibustion, etc. These therapies have been used for managing headache including migraine for centuries in China [23, 24], and have been recorded in Chinese medical classical literature. In recent decades, CM therapies become increasingly popular worldwide [25-27]. A recently published research analysed the health insurance data in China and reported that, CM therapies were applied in more than 60% of migraine-related outpatient visits, which is equivalent with the usage of western medications [28].

Research on effectiveness of acupuncture for migraine has been conducted globally and published in international journals. Latest systematic reviews since 2019 indicate that acupuncture is an effective modality for episodic migraine [29-34]. But the only available systematic review summarising the effectiveness of acupuncture for menstrual migraine provided negative results [35]. There has not been any systematic review evaluating acupuncture for chronic migraine, but a small-sized RCT reported that acupuncture achieved better effectiveness and caused less adverse events compared to botulinum toxin A injection for chronic migraine [36]. In addition, a retrospective cohort study reported that, acupuncture reduced medical expenditure and the risk of depression and anxiety during the long-term follow-up period in migraine patients [37]. In summary, acupuncture is evidenced as an effective therapy for episodic migraine, but there is insufficient evidence supporting the treatment effects of acupuncture for chronic migraine, menstrual migraine and the comorbidities of migraine.

Systematic reviews published in Chinese language indicated that, CHM presented a promising benefit for migraine both as a prophylactic therapy and pain relief [38-42]. However, the certainty of evidence was downgraded due to the low methodological quality and small sample size. Only one systematic review published in English language was based on high quality RCTs and highlighted the effectiveness of chuanxiong formulae for migraine [43]. In addition, some clinical trials revealed that CHM benefit menstrual migraine [44-46], but evidence of CHM for chronic migraine is limited. On the contrary, experimental evidence of CHM has been well established. The mechanism of certain herbal extracts for migraine has been thoroughly investigated [47-49]. Therefore, rigorously designed clinical research on CHM for migraine are needed for promoting the evidence internationally.

Limited generalisability of existing evidence and insufficient real-world evidence regarding Chinese medicine therapies for migraine management

It should be pointed out that, applying personalised and tailored treatment based on individual’s syndrome differentiation, is the core characteristic of CM [7]. Following the syndrome differentiation approach, patients suffering from the same disease may be treated with different herbs or acupuncture points, and it is also possible that patients suffering from different diseases may be treated with the same herbs or acupuncture points.

Current clinical research evidence of CM therapies on migraine is mainly generated from RCTs, which required strict selection criteria and applied unified treatment methods [8]. Results of these RCTs might have failed to reflect the real practice of CM therapies, particularly among migraineurs who often suffer from more complex clinical conditions and receive dynamic treatments. Such indirectness and limited external validity of evidence from previous explanatory RCTs might had weaken the certainty of the evidence [8]. Real-world evidence in medicine means evidence obtained from real world data, which are observational data obtained outside the context of RCTs and generated during routine clinical practice [50], which could be supplementary evidence to those being provided by RCTs [51, 52]. Clinical data collected from medical record and patient-reported questionnaires during routine clinical practice, is a promising approach for generating clinical evidence, summarising clinical knowledge, and providing valuable information for clinical decision making, particularly for CM therapies [53].

To provide more realistic, practical and convincing evidence of CM for migraine management, evidence based on real-world studies are needed.

Evidence based practice and shared medical decision become leading trend in clinical practice

Evidence based practice emphasises that decisions about health and social care are based on the best available, current, valid and relevant evidence. These decisions should be made by those receiving care, informed by the tacit and explicit knowledge of those providing care, within the context of available resources [54].

Shared decision-making means that decisions are shared by doctor and patient and informed by best evidence, not only about risks and benefits but also patient specific characteristics and values. There are three vital and equally important elements: the expertise of the provider, the medical evidence, and the patient’s preferences. Shared decision making is the process of integrating patient’s goals and concerns with medical evidence to achieve high-quality medical decisions [55].

As reported in previous research, there are three common concerns raised by migraine patients: Firstly, quality of life issues around coping with pain, sleep disturbance and restriction on daily activities, education, working and social life, and the impact it has on the family. Secondly, concerns around side effects of pharmacological therapies, medication overuse and feeling dependent on prophylactic therapies. Thirdly, the need for clear information on the use of preventer medication [13].

In this study, migraine patients’ concerns will be addressed through relevant outcome measures. Hence the study will formulate an all-sided evidence of CM for migraine, in dimensions of experts’ experience, research evidence and patients’ goals. This will strongly support the evidence-based practice and clinical decision making, therefore benefit the migraineurs in a greater manner.

1. Objectives and research questions

Objectives

This study is to generate research evidence of effectiveness and safety of CM therapies for migraine and to summarise regularity and utilisation of CM for migraine.

Research questions

Research question 1

What is the real situation and utilisation patterns of CM therapies applied for migraine in clinical practice based on a CM hospital?

Research question 2

What are the effectiveness and safety of CM therapies for migraine? What are the factors resulting in different effectiveness among migraine patients? What are the concerns and preferences of the migraine patients when seeking CM for migraine?

1. Methodologies

This research project is designed as two components to answer the above-mentioned research questions, detailed methodology of each section is presented below.

Section 1: Utilisation and prescription patterns of Chinese medicine for migraine: a retrospective study based on electronic medical records

Research aim

The retrospective study is to summarise and analyse the utilisation regularity and prescription patterns of CM for migraine, based on electronic medical records of GPHCM.

Design

The real-world study will be retrospectively conducted based on electronic medical records with migraine being the primary diagnosis, from the out-patient department of GPHCM between 1^st^ July 2018 and 1^st^ July 2020. The researcher is not accessible to the identifiable information such as the names, identification card number, home address, birthday, contact number, etc. Therefore, it is not possible for the researcher to gain consent from the patients. This section of study will not involve confidential information and there is no anticipated harms or risks. A waiver of consent is to be applied.

Eligibility criteria

#### Inclusion criteria

- Migraine being the primary diagnosis.
- Medical records with detailed information of treatment.

#### Exclusion criteria

- Migraine-like headache attacks induced by or comorbid with brain injury, brain tumour, glaucoma, and other diseases.
- Pregnant females.
- The medical visits aimed for purchasing prescription medicine other than migraine related medicine.
- Main complains not related with migraine.
- Descriptions of headache not compliance to diagnosis criteria of migraine in ICHD-3.

Data collection and preparation

#### Exporting data

All the electronic medical records will be exported from the medical system, in form of Word files separately by the Information Technology department of GPHCM.

#### Screening for eligibility

The diagnoses and treatment information will be screened to exclude the ineligible ones with reasons recorded, according to the exclusion criteria.

#### Split and classify the information

For the eligible medical records, the visiting date, medical card number, gender, age, geographic location, departments and professional title of doctors the patients visited, chief complains, current history of migraine, previous medical diagnosis, allergic history, family history of migraine, menstrual cycle information (if available), CM diagnosis along with CM syndrome differentiation, names, dosage and administration methods of prescriptions (both CM and western medicine prescriptions), will be extracted and exported in Excel Spreadsheet, in chronological order of the medical visits of each patient.

#### Further extraction

Further information such as comorbid symptoms including sleeping disorders, anxiety and depression, characteristic symptoms like nausea, vomiting, photophobia, etc, detailed information of migraine such as the disease duration, pain intensity and attack frequency, onset age, analgesic consumptions, treatment feedbacks will also be extracted. Visit frequency and treatment course of each patient will be counted and recorded. Additionally, those records indicating improvement in migraine symptom will be highlighted. Reduction in pain severity, pain frequency and drug consumption will be marked.

Each individual commercial Chinese medicine product will be recorded in a separate column, followed by detailed ingredients of the product. Each single herb of an herbal formula or acupuncture point from an acupuncture prescription will also be entered in separate columns.

#### Standardisation

During the procedure of data transformation, herbs with several names will be unified according to Chinese Materia medica [56] and Chinese Pharmacopoeia [57]. While herbs processed with different methods will be recorded and stored as their original names.

Privacy protection

The digital data will be stored on password-locked computer drive and stored for 5 years.

Patients’ names will be masked by codes, which are composed of their initials and the last 4-digid number of their medical card. And the identifiable information like the full name, medical card number, phone number, will be removed from the dataset.

All the procedures mentioned above will be conducted and completed by the named researchers in the ethics application.

Statistical analysis

Principally, quantitative variables such as age, migraine frequency, disease courses, treatment courses, etc., will be calculated as means and standard deviations (SD) to present the characteristics of the patients. The above variables will be compared between groups using students’ test or analysis of variance (ANOVA) if the data is normally distributed. Quantitative variables that do not distribute normally will be displayed as medium and interquartile range and compared between groups using the rank sum test. Categorical variables such as subtypes of migraine, treatment methods, etc, will be calculated as numbers and percentages to demonstrate the treatment regularity of CM, and they will be compared between groups using Pearson’s chi-square or Fisher’s exact test, when applicable. A p value of <0.05 will be considered significant. Frequency count, cluster analysis, Apriori Algorithm will be applied to present the prescription patterns. Multivariate regression analysis will be applied to explore and identify predictors for treatment patterns, and predictive demographic characteristic for clinical manifestations. Medical records reporting prognosis of the disease, comorbidities like insomnia and depression, analgesic consumption, or subtype of migraine like chronic migraine and menstrual migraine, will be filtered for further analyse. The analyses will be conducted using SPSS 26 [58].

Ethical considerations

The retrospective study is based on existing medical records. Confidential information such as names, birthdays, identification card numbers, home addresses and contact information are not accessible. No identifiable information will be used in this study. There are no anticipated harms of health, reputation, psychology and economic for the patients.

Section 2: Chinese herbal medicine for migraine management: a prospective, registry, cohort study in a real-world setting

Research aim

Based on real-world setting, the project is to summarise the common patterns of CHM for migraine management. Secondly, the study is to explore the relationship between CHM exposure with clinical response, particularly the analgesics consumption, and detect the predictive factors for clinical improvements. Furthermore, this section will address migraineurs’ concerns regarding CM. The results of this project will involve the three vital elements of evidence-based practice: expert experience being summarised from the regularity of prescriptions, patients’ preferences obtained from the survey about their concerns and related outcomes, and clinical evidence generated by the cohort study. CHM is being investigated because of its predominant role for migraine management according to previous medical records.

Study design

The prospective registry study on migraine will be conducted in the Outpatient Headache Clinic at GPHCM, from December 2020 to May 2022.

Recruitment advertisements will be posted in the waiting room of the headache clinic of GPHCM. Screening forms will be distributed to the headache specialists after they received training for this observational study. Newly diagnosed migraine patients will be further evaluated and fully informed by the researcher in a private room. Written informed consents are required. Subjects who refuse to be observed will get fair medical managements and the enrolled participants could withdraw from the observation anytime. Particularly, migraine patients visiting the researcher (S. Lyu) for medical managements will be informed and recruited by an independent research assistant. The decision to participate in the research should be supported by a third party.

The participants will be followed up on a four-weeks basis over 12 weeks after commencing treatment. Data including demographic information, assessments of migraine and the common comorbidities will be collected by questionnaires at baseline and three follow-up time points (Week 4, Week 8, and Week 12). Outcomes will be compared among sub-cohorts grouped according to different criteria, for example, treatment courses, treatment methods they received and other clinical characteristics, etc. The procedure of this study is presented in Figure 1.


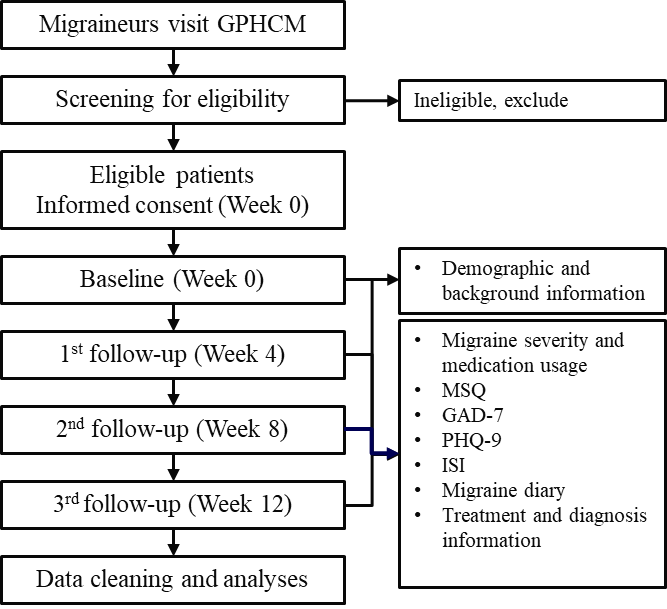


Figure 1 Flow Chart of Cohort Study Procedure

Note: GAD-7: Generalised Anxiety Disorder 7-item Scale, GPHCM: Guangdong Provincial Hospital of Chinese Medicine, ISI: Insomnia Severity Index Scale, MSQ: Migraine Specific Questionnaire, PHQ-9: Patient Health Questionnaire-9.

Eligibility criteria

#### Inclusion criteria

Eligible participants should meet all the following criteria:

1. Subjects being primarily diagnosed with migraine according to ICHD-3 [1].
2. Subjects seeking treatments for migraine in GPHCM for the first time.
3. Agreed and signed the informed content.
4. Aged above 18.
5. Not concurrently participating in other intervention trials.

#### Exclusion criteria

Meeting any of the following criteria, the subject will not be registered. The number of participants being excluded will be recorded.

1. Subjects ever diagnosed with diseases which could induce migraine-like headache such as glaucoma, brain tumour, brain injury. Medication overuse headache reattributed to migraine is allowed.
2. Subjects with severe linguistic, visual, mental, memory or cognitive impairment and incapable to complete the questionnaire.
3. Pregnant or lactating females.
4. Subjects highly dependent on medical managements.

#### Drop-out criteria

Drop-outs will be marked in the following situations:

1. The diagnosis of migraine is recorrected as secondary headache during the observation procedure.
2. The participants request to withdraw from the observation.
3. The participants do not complete the 12-weeks follow-up for any reason.

Data collected from participants in the latter two situations will still be analysed. Number of dropouts will be recorded, and the reasons will be summarised, if available.

Demographic information

Demographic information including gender and age, background information like migraine course, family history, migraine subtypes and medical histories etc. will be collected at entrance (Week 0). Patient’s concerns and preferences will also be surveyed at entrance. This data will be collected via structured questionnaires and then be entered into Excel sheet. Data checking will be conducted promptly. Missing data will be filled in after contacting the participants for correct answers. See appendix for detailed information.

Observational variables

#### Outcomes

According to clinical trial design guidelines for migraine [59] and the research purposes, the outcome measures are defined as follows and could be collected questionnaires in the appendix:

Migraine frequency (migraine attacks per four weeks), responder rate (response is defined as 50% drop in migraine frequency after four weeks), migraine days (days with migraine headache per four weeks), migraine duration (average hours of migraine attacks), NRS, analgesic consumption (using medication quantification scale) [60], Migraine Specific Quality of Life Questionnaire (MSQ) [61], Generalised Anxiety Disorder 7-item Scale (GAD-7) [62], Patient Health Questionnaire-9 (PHQ-9) [63], Insomnia Severity Index Scale (ISI) [64, 65], 5-point Likert scale on satisfaction of the treatment. High scores of PHQ-9 will be reported to the clinicians for further medical managements, since the higher the score of PHQ-9, the higher risk of suicide the subject would experience.

Adverse events: Any possible or suspicious adverse events throughout the study, such as diarrhea, dental ulcer, insomnia, etc, will be recorded in the migraine diary.

#### Treatment and diagnosis information

Treatment information including details of prescriptions will be derived from electronic medical record systems along with the current and history diagnoses. (See Appendix).

Data collection via questionnaires at different timepoints are summarised in Table 1. For those who failed to complete the questionnaires by themselves, help will be provided by the researcher.

Table 1 Questionnaires for different timepoints

|  | Demographic and background information | Migraine severity and medication usage | MSQ | GAD-7 | PHQ-9 | ISI | Treatment and diagnosis information | Migraine diary |
| --- | --- | --- | --- | --- | --- | --- | --- | --- |
| Baseline (Week 0) | √ | √ | √ | √ | √ | √ | √ | √ |
| 1^st^ follow up (Week 4) |  | √ | √ | √ | √ | √ | √ | √ |
| 2^nd^ follow up (Week 8) |  | √ | √ | √ | √ | √ | √ | √ |
| 3^rd^ follow up (Week 12) |  | √ | √ | √ | √ | √ | √ | √ |

Note: GAD-7: Generalised Anxiety Disorder 7-item Scale, ISI: Insomnia Severity Index Scale, MSQ: Migraine Specific Quality of Life Questionnaire, PHQ-9: Patient Health Questionnaire-9.

Sampling method and sample size

This study aims to explore the information available based on a registry of patient cases from GPHCM available in a specific time range, sample size target is not required since there are no pre-determined hypotheses to be tested. For the registry study, consecutive sampling method will be used to recruit participants to reduce the selection bias for non-randomised studies [66]. In this project, patients’ data will be collected for a full year, the estimated number of participants is approximately 400 according to the average outpatient visits

Potential exposures for sub-cohort analysis

Sub-cohort analysis is proposed to be conducted based on the treatment methods, treatment courses, subtypes of migraine (episodic or chronic migraine, menstrual or non-menstrual migraine), comorbidities of migraine (according to the scores of GAD-7, PHQ-9 and ISI), baseline characteristics of migraine (course of migraine, average frequency, average pain NRS and analgesics consumption at entrance).

Privacy protection

The research data will be stored in password-protected cloud drive in RMIT University and GPHCM for at least five years. Only research members could access to the data for research purpose. In cases of severe adverse events, the ethics committee member could access the data for safety.

Statistical analysis

#### Analytical methods

Categorical variables such as gender and subtypes of migraine, will be presented using frequencies and percentages, and quantitative variables such as disease course, migraine frequency and pain severity will be displayed using standard measures of central tendency and dispersion, with mean and SD if data is normally distributed. Data not normally distributed will be described with median or inter-quartile ranges.

T-test, ANOVA or rank sum test will be used to compare quantitative variables between cohorts after testing for normality. Chi-square or Fisher’s exact test will be performed for categorical variables to test differences between cohorts. All the *p* values will be two-tailed at the 95% significance level.

Multiple correlation and multivariable regression analysis will be conducted to explore the predictors of clinical improvements, if available. Repeated measure ANOVA will be applied to evaluate the differences of quantitative dependent variables such as migraine frequency, among sub-cohorts at various follow-up endpoints, while generalised estimated equation will be adopted for categorical dependent variables and those do not meet the criteria of repeated measure ANOVA.

All statistical analyses will be performed using SPSS 26.

#### Control for potential bias

At the recruiting stage, consecutive sampling methods will be adopted to reduce the selection bias and eligible criteria will be applied to reduce the bias of misdiagnoses.

During the observation period, migraine diary in monthly bases will be allocated to the participants to reduce the potential recall bias during follow-up timepoints.

When individuals are followed-up over an extended period, it is inevitable that measurements on particular variables are sometimes missing. Various methods of data collection will be introduced during the follow-up period. Reminders will be sent via Wechat or phone message to the patients before the follow-up timepoint to improve participants’ compliance. An independent data monitoring team will conduct data checking regularly to detect missing data during the study, and the chief investigator will remind participants promptly through phone calls or Wechat.

Ethics considerations

The participants might experience a minimal phycological discomfort and inconvenience during the observation. The researcher communicates with participants respectfully to minimise any discomfort associated with the study and provide multi choices of follow-up methods for their conveniences. No extra economic costs nor additional physical or social harms are anticipated in this observational study, no payments will be provided to the participants.

Thought the subjects will not receive direct benefits for their participants in this study, they will get a comprehensive assessment of migraine comorbidities, a monthly summary and feedback of their migraine conditions. The findings of this study will hopefully provide practical evidence for future clinical practice for migraine and therefore benefit a larger range of migraine patients.

The data will be confidentiality maintained. All identifiable electronic data are kept in password-protected cloud drive, and the paper documents are stored in locked cabinets in a secured, private office. The contact information will only be accessible to the researchers for research purpose. The data will be deidentified prior to being analysed.

1. Expected deliverables

- CM therapies used in clinical practice for migraine will be summarised based on data mining in real-world medical records.
- The results from a prospective cohort study will provide evidence on CM therapies’ effectiveness and safety. Patients’ preference, goals and concerns related to CM therapies will also be summarised for better clinical decision making.

1. Significance

Migraine is a clinical difficult and complicated disease, and there is no cure for it at present. Migraine is associated with complex comorbidities and potential risk of medication overuse. There have been certain clinical evidence supporting CM therapies, including acupuncture and CHM, for the management of migraine. However, the current research evidence does not address the features of individual-tailored CM therapies utilised in migraine clinical management.

This project is designed to summarise clinicians’ experience and to generate research evidence through the cohort clinical study. Patients’ preference in terms of quality of life, migraine associated comorbidities and medication usage issue will also be addressed by the prospective cohort study. In summary, this study will provide all-sided evidence of CM therapies, therefore assist evidence-based practice and shared decision making in clinical management of migraine.

1. Research schedule

**Table 2: Timeline of the research**

| Items | Time period | Items | Time period |
| --- | --- | --- | --- |
| Prospective cohort study | | Retrospective study based on medical records | |
| Ethics application in GPHCM | 2020.10.25-2020.11.30 | Ethics application in GPHCM | 2020.10.25-2020.11.30 |
| Patients’ recruitment | 2020.12.01-2022.02.28 | Exporting medical records | 2020.12.01-2020.12.15 |
| Follow-ups | 2020.12.01-2022.05.31 | Data management | 2020.12.16-2021.02.28 |
| Data management | 2022.03.01-2022.06.30 | Report writing | 2021.03.01-2021.06.30 |
| Report writing | 2022.07.01-2022.09.30 | Publishing | 2021.07.01-2021.12.30 |
| Publishing | 2022.10.01-2022.12.30 |  |  |

1. Research member list and assignments

| Names | Institution | Education degree | Assignments | Conflict of interest |
| --- | --- | --- | --- | --- |
| Shaohua Lyu | GPHCM, RMIT University | Master of medicine | Design and conduct the study.  Recruit, evaluate and follow up the participants.  Collect and analyse data.  Report the study results. | No |
| Xinfeng Guo | GPHCM | PhD | Direct the design of study, monitor the data quality. | No |
| Jingbo Sun | GPHCM | PhD | Help to communicate and coordinate during the whole procedure. Monitor the data quality. | No |
| Charlie Changli Xue | RMIT University | PhD | Direct the design of study | No |
| Anthony Lin Zhang | RMIT University | PhD | Direct the design and conduction of study, as well as the data management and analyses. | No |
| Claire Shuiqing Zhang | RMIT University | PhD | Direct the design and conduction of study, as well as the data management and analyses. | No |
| Zhenhui Mao | GPHCM | Bachelor of Medicine | Assist the researcher in participants recruitment, data collection, follow-ups. | No |

1. Appendix

Questionnaire 1: Demographic and Background Information

Questionnaire 2: Migraine Severity and Medication Usage at entrance

Questionnaire 3: Migraine Specific Quality of Life Questionnaire (MSQ)

Questionnaire 4: Generalized Anxiety Disorder 7-item scale (GAD-7)

Questionnaire 5: Patient Health Questionnaire-9 (PHQ-9)

Questionnaire 6: Insomnia Severity Index (ISI)

Diagnosis and Treatment Information

Migraine Diary

Questionnaire 7: Migraine Severity and Medication Usage for Follow-up Visits

1. References

[1] HEADACHE CLASSIFICATION COMMITTEE OF THE INTERNATIONAL HEADACHE SOCIETY (IHS). The International Classification of Headache Disorders 3rd edition [J]. Cephalalgia, 2018, Jan;38(1):1-211.(

[2] LIPTON R B, BUSE D C, SERRANO D, et al. Examination of unmet treatment needs among persons with episodic migraine: results of the American Migraine Prevalence and Prevention (AMPP) Study [J]. Headache, 2013, 53(8): 1300-11.

[3] YOUNG N P, PHILPOT L M, VIERKANT R A, et al. Episodic and Chronic Migraine in Primary Care [J]. Headache, 2019, 59(7): 1042-51.

[4] FINOCCHI C, VILLANI V, CASUCCI G. Therapeutic strategies in migraine patients with mood and anxiety disorders: clinical evidence [J]. Neurol Sci, 2010, 31 Suppl 1(S95-8.

[5] DIENER H C, HOLLE D, SOLBACH K, et al. Medication-overuse headache: risk factors, pathophysiology and management [J]. Nature reviews Neurology, 2016, 12(10): 575-83.

[6] MINEN M T, BEGASSE DE DHAEM O, KROON VAN DIEST A, et al. Migraine and its psychiatric comorbidities [J]. Journal of neurology, neurosurgery, and psychiatry, 2016, 87(7): 741-9.

[7] CAO H. Basic theory of traditional Chinese medicine [M]. Beijing: China Press of Traditional Chinese Medicine, 2004.

[8] BLACK N. Why we need observational studies to evaluate the effectiveness of health care [J]. BMJ (Clinical research ed), 1996, 312(7040): 1215-8.

[9] DELOITTE ACCESS ECONOMICS. Migraine in Australia Whitepaper [M]. 2018.

[10] STEINER T J, STOVNER L J, VOS T. GBD 2015: migraine is the third cause of disability in under 50s [J]. J Headache Pain, 2016, 17(1): 104.

[11] YAO C, WANG Y, WANG L, et al. Burden of headache disorders in China, 1990-2017: findings from the Global Burden of Disease Study 2017 [J]. J Headache Pain, 2019, 20(1): 102.

[12] CEPHALALGIA GROUP PAIN-RELIEVING BRANCH CHINESE MEDICAL ASSOCIATION. Guideline for migraine in China [J]. Chinese Journal of Pain Medicine, 2016, 22(10):

[13] SIGN 155 • Pharmacological management of migraine [M]. 2018.

[14] MARCI CLARK T J S, STEWART J TEPPER, NIMANEE HARRIS,SUSAN MARTIN, SANDHYA SAPRA , NEEL SHAH. Patient satisfaction with prophylactic migraine medications [M]. the 59th Annual Scientific Meeting American Headache Society. Boston, MA. 2017.

[15] PEER MOHAMED B, GOADSBY P J, PRABHAKAR P. Safety and efficacy of flunarizine in childhood migraine: 11 years' experience, with emphasis on its effect in hemiplegic migraine [J]. Developmental medicine and child neurology, 2012, 54(3): 274-7.

[16] PARDUTZ A, SCHOENEN J. NSAIDs in the Acute Treatment of Migraine: A Review of Clinical and Experimental Data [J]. Pharmaceuticals (Basel), 2010, 3(6): 1966-87.

[17] TORTA R, IERACI V. Migraine and depression comorbidity: antidepressant options [J]. Neurol Sci, 2012, 33 Suppl 1(S117-8.

[18] VETVIK K G, MACGREGOR E A. Sex differences in the epidemiology, clinical features, and pathophysiology of migraine [J]. The Lancet Neurology, 2017, 16(1): 76-87.

[19] KOZAK H H, BOYSAN M, UCA A U, et al. Sleep quality, morningness-eveningness preference, mood profile, and levels of serum melatonin in migraine patients_ a case-control study [J]. Acta neurologica Belgica, 2017, 117(1): 111-9.

[20] ALSTADHAUG K S R, BEKKELUND S. Insomnia and circadian variation of attacks in episodic migraine [J]. Headache, 2007, 47(8): 1184-8.

[21] JIYOUNG KIM S-J C, WON-JOO KIM, KWANG IK YANG, CHANG-HO YUN AND MIN KYUNG CHU. Insufficient sleep is prevalent among migraineurs: a population-based study [J]. The Journal of Headache and Pain, 2017, 18(1): 50.

[22] VERSPEELT J, DE LOCHT P, AMERY W K. Post-marketing cohort study comparing the safety and efficacy of flunarizine and propranolol in the prophylaxis of migraine [J]. Cephalalgia : an international journal of headache, 1996, 16(5): 328-36; discussion 288.

[23] ANJI LIN Y W. Chinese medicine for 'Toufeng': a literature review; proceedings of the The first International Forum on the Development of Traditional Chinese Medicine, the First International Symposium on the Prevention and Treatment of AIDS by Traditional Chinese Medicine, and the Association of Directors of National Scientific Research Institutes of Traditional Chinese Medicine, Beijing China, F, 2005 [C].

[24] WANG W. The regularition of Chinese medicine for migraine: report based on medical records [D]; Guangzhou University of Traditional Chinese Medicine, 2017.

[25] ITALIA S, BRAND H, HEINRICH J, et al. Utilization of complementary and alternative medicine (CAM) among children from a German birth cohort (GINIplus): patterns, costs, and trends of use [J]. BMC complementary and alternative medicine, 2015, 15(49.

[26] YANG L, ADAMS J, SIBBRITT D. Prevalence and factors associated with the use of acupuncture and Chinese medicine: results of a nationally representative survey of 17161 Australian women [J]. Acupuncture in medicine : journal of the British Medical Acupuncture Society, 2017, 35(3): 189-99.

[27] DE MORAES MELLO BOCCOLINI P, SIQUEIRA BOCCOLINI C. Prevalence of complementary and alternative medicine (CAM) use in Brazil [J]. BMC Complement Med Ther, 2020, 20(1): 51.

[28] YU S, ZHANG Y, YAO Y, et al. Migraine treatment and healthcare costs: retrospective analysis of the China Health Insurance Research Association (CHIRA) database [J]. J Headache Pain, 2020, 21(1): 53.

[29] ZHANG N, HOULE T, HINDIYEH N, et al. Systematic Review: Acupuncture vs Standard Pharmacological Therapy for Migraine Prevention [J]. Headache, 2020, 60(2): 309-17.

[30] CHEN Y Y, LI J, CHEN M, et al. Acupuncture versus propranolol in migraine prophylaxis: an indirect treatment comparison meta-analysis [J]. Journal of neurology, 2020, 267(1): 14-25.

[31] LI Y X, XIAO X L, ZHONG D L, et al. Effectiveness and Safety of Acupuncture for Migraine: An Overview of Systematic Reviews [J]. Pain research & management, 2020, 2020(3825617.

[32] TRINH K V, DIEP D, CHEN K J Q. Systematic Review of Episodic Migraine Prophylaxis: Efficacy of Conventional Treatments Used in Comparisons with Acupuncture [J]. Medical acupuncture, 2019, 31(2): 85-97.

[33] LI X, DAI Q, SHI Z, et al. Clinical Efficacy and Safety of Electroacupuncture in Migraine Treatment: A Systematic Review and Network Meta-Analysis [J]. The American journal of Chinese medicine, 2019, 47(8): 1755-80.

[34] ZHANG X T, LI X Y, ZHAO C, et al. An Overview of Systematic Reviews of Randomized Controlled Trials on Acupuncture Treating Migraine [J]. Pain research & management, 2019, 2019(5930627.

[35] YANG M, DU T, LONG H, et al. Acupuncture for menstrual migraine: a systematic review [J]. BMJ supportive & palliative care, 2020,

[36] NADERINABI B, SABERI A, HASHEMI M, et al. Acupuncture and botulinum toxin A injection in the treatment of chronic migraine: A randomized controlled study [J]. Caspian journal of internal medicine, 2017, 8(3): 196-204.

[37] LIAO C C, LIAO K R, LIN C L, et al. Long-Term Effect of Acupuncture on the Medical Expenditure and Risk of Depression and Anxiety in Migraine Patients: A Retrospective Cohort Study [J]. Frontiers in neurology, 2020, 11(321.

[38] JUENING YAO B Z, KEGANG CAO. Effect of external application of Chinese medicine on acupoint for migraine: a systematic review based on RCTs [J]. World Traditional Chinese Medicine, 2019, 14(11): 2930-4.

[39] WEIDONG LUO J W, YAXIAN CAI, GUOHUA CHEN. Effectiveness and safert of intergrated Chinese medicine and western medicine for migraine: a systematic review and meta-analysis [J]. Hunan Journal of Traditional Chinese Medicine, 2018, 34(07): 157-60.

[40] YINHE CAI K L, WEIPENG SUN, ZHIBING WU. Effectiveness of Tianmagouteng formula compared with CCB for migraine: a systematic review and meta-analysis [J]. Chinese Journal of Basic Medicine of TCM, 2018, 24(07): 949-54.

[41] MENGUO YUAN Y L, TING TIAN, WEIFENG GUO. Effectiveness of the method of activating blood and removing wind for migraine: a systematic review and meta-analysis [J]. Guiding Journal of Tradication Chinese Medicine, 2017, 23(01): 73-5+9.

[42] XIAOWEN YU G L, ZHONGLIN WANG. Effectiveness of Tongqiaohuoxue formular for migraine: a systematic review based on RCTs [J]. Journal of Shandong University of Chinese Medicine, 2017, 41(03): 202-6.

[43] SHAN C S, XU Q Q, SHI Y H, et al. Chuanxiong Formulae for Migraine: A Systematic Review and Meta-Analysis of High-Quality Randomized Controlled Trials [J]. Frontiers in pharmacology, 2018, 9(589.

[44] SHOURAN LI W N, JIN WEN. Effect of Chaihuguizhi formular for menstrual migraine: a clinical trial [J]. Jilin Journal of Tradication Chinese Medicine, 2018, 38(9):

[45] DUAN Z. Case series of Tongqiaohuoxue Formula for menstrual migraine [J]. Henan Journal of Chinese medicine, 2013, 33(11):

[46] SHUN XIU WU C Y X, XIAN GUANG CHEN, SEN MEI LI, LI LING WEI. Injection of Yimucao for menstrual migraine: a clinical trial [J]. Liaoning Journal of Traditional Chinese Medicine, 2004, 31(12):

[47] LAI T, CHEN L, CHEN X, et al. Rhynchophylline attenuates migraine in trigeminal nucleus caudalis in nitroglycerin-induced rat model by inhibiting MAPK/NF-кB signaling [J]. Mol Cell Biochem, 2019, 461(1-2): 205-12.

[48] LIU Z K, NG C F, SHIU H T, et al. Neuroprotective effect of Da Chuanxiong Formula against cognitive and motor deficits in a rat controlled cortical impact model of traumatic brain injury [J]. Journal of ethnopharmacology, 2018, 217(11-22.

[49] GUAN J, ZHANG X, FENG B, et al. Simultaneous determination of ferulic acid and gastrodin of Tianshu Capsule in rat plasma by ultra-fast liquid chromatography with tandem mass spectrometry and its application to a comparative pharmacokinetic study in normal and migraine rats [J]. J Sep Sci, 2017, 40(21): 4120-7.

[50] GARRISON JR L P, NEUMANN P J, ERICKSON P, et al. Using real‐world data for coverage and payment decisions: The ISPOR real‐world data task force report [J]. Value in health, 2007, 10(5): 326-35.

[51] SHERMAN R E, ANDERSON S A, DAL PAN G J, et al. Real-World Evidence - What Is It and What Can It Tell Us? [J]. N Engl J Med, 2016, 375(23): 2293-7.

[52] GROOTENDORST D C, JAGER K J, ZOCCALI C, et al. Observational studies are complementary to randomized controlled trials [J]. Nephron Clin Pract, 2010, 114(3): c173-7.

[53] LIU B, ZHOU X, WANG Y, et al. Data processing and analysis in real-world traditional Chinese medicine clinical data: challenges and approaches [J]. Stat Med, 2012, 31(7): 653-60.

[54] DAWES M, SUMMERSKILL W, GLASZIOU P, et al. Sicily statement on evidence-based practice [J]. BMC Med Educ, 2005, 5(1): 1.

[55] ALSTON C, PAGET L, HALVORSON G, et al. Communicating with patients on health care evidence [J]. NAM Perspectives, 2012,

[56] XUEMIN G. Chinese materia medica [M]. China Press of Traditional Chinese Medicine, 2000.

[57] STATE PHARMACOPOEIA COMMITTEE OF CHINA. Chinese Pharmacopoeia [M]. 2015.

[58] IBM CORP. IBM SPSS Statistics for Windows [M]. Armonk, NY; IBM Corp. 2017.

[59] TFELT-HANSEN P, PASCUAL J, RAMADAN N, et al. Guidelines for controlled trials of drugs in migraine: third edition. A guide for investigators [J]. Cephalalgia, 2012, 32(1): 6-38.

[60] HARDEN R N, WEINLAND S R, REMBLE T A, et al. Medication Quantification Scale Version III: update in medication classes and revised detriment weights by survey of American Pain Society Physicians [J]. The journal of pain : official journal of the American Pain Society, 2005, 6(6): 364-71.

[61] MARTIN B C, PATHAK D S, SHARFMAN M I, et al. Validity and reliability of the migraine‐specific quality of life questionnaire (MSQ Version 2.1) [J]. Headache: The Journal of Head and Face Pain, 2000, 40(3): 204-16.

[62] SPITZER R L, KROENKE K, WILLIAMS J B W, et al. A Brief Measure for Assessing Generalized Anxiety Disorder: The GAD-7 [J]. Archives of Internal Medicine, 2006, 166(10): 1092-7.

[63] KROENKE K, SPITZER R L, WILLIAMS J B. The PHQ-9: validity of a brief depression severity measure [J]. J Gen Intern Med, 2001, 16(9): 606-13.

[64] MORIN C M, BELLEVILLE G, BÉLANGER L, et al. The Insomnia Severity Index: Psychometric Indicators to Detect Insomnia Cases and Evaluate Treatment Response [J]. Sleep, 2011, 34(5): 601-8.

[65] BAGHYAHI S B A, GAO Y, TAGHANAKI H B, et al. 2738–Reliability and validity of the chinese translation of insomnia severity index (C-ISI) in chinese patients with insomnia [J]. European Psychiatry, 2013, 28(S1): 1-.

[66] GLIKLICH RE D N, LEAVY MB. Registries for Evaluating Patient Outcomes: A User's Guide [M/OL]. April 2014[<https://www.ncbi.nlm.nih.gov/books/NBK208632/>.
