# Supplementary material for: Migraine patients visiting Chinese medicine hospital: Protocol for a prospective, registry-based, real-world observational cohort study
Source: PLoS One. 2022 Mar 15;17(3):e0265137. doi: 10.1371/journal.pone.0265137 (PMC8923465; doi:10.1371/journal.pone.0265137)
Supplement: Attachment — Submitted filename: Response to Reviewers.docx [file pone.0265137.s006.docx]

Dear Prof. Sarah Michiels,

We would like to express our greatest appreciation to you and the reviewers for all comments on the manuscript entitled “Migraine Patients Visiting Chinese Medicine Hospital: Protocol for a Prospective, Registry-Based, Real-World Observational Cohort Study” (PONE-D-21-25486). We have carefully considered the comments and amended the manuscript accordingly. We sincerely hope that the revised manuscript will meet the requirement of PLOS ONE, and therefore it will be considered for publication.

The responses to comments are listed as following:

# Comments from the Editor:

1. Please ensure that your manuscript meets PLOS ONE's style requirements, including those for file naming.

Response: The manuscript was resubmitted in compliance with PLOS ONE’s style requirements.

2. We note that the grant information you provided in the “Funding Information” and “Financial Disclosure” sections do not match.

When you resubmit, please ensure that you provide the correct grant numbers for the awards you received for your study in the ‘Funding Information’ section.

Response: Thanks for pointing this out. We have checked the funding information and made them consistent.

3. Your ethics statement should only appear in the Methods section of your manuscript. If your ethics statement is written in any section besides the Methods, please delete it from any other section.

Response: The ethics statement written in sections other than the Methods (Line 113-114) has been deleted.

# Comments from Reviewer #1

1. The step from RCT to observational trials to address all issues within an RCT-design is rather drastic, and the existence of pragmatic trials is ignored. I would suggest the authors to consider a pragmatic trial or - in case a pragmatic trial is infeasible - I would suggest the authors to focus more on patterns in healthcare behaviour.

2. Inferring on the effectiveness of CHM (as stated in the objectives of the authors "evaluate the effectiveness and safety of individualized CHM for adult migraine in a real-world setting;" will be a tough - if not impossible - challenge based on the method that was presented in the protocol of the authors. I would suggest the authors to consider creating a DAG, in which they represent the theoretical background on how the study might infer on a causal relation between exposure-outcome.

Response to comments 1&2:

Thanks for your comments and suggestions.

We revised the objectives of this study (see Line 94-99).

This study is an observational study, without any intervention being provided by the researcher. The purpose of this study is to explore the effects and safety of CHM treatments in the real-world clinical practice. Therefore, there is no causal inference would be made based on our study results.

However, the findings from this observational study will be invaluable for the future design of a pragmatic trial.

3. In the data-analyses, the authors state "Multiple imputation will be carried out to deal with missing data". However, there is no information on the methodology that will be used to reach this goal. Which packages will be used?
Why do you rely on the Missing at Random (MAR) hypothesis? What if the data is rather MNAR?

Response:

In this observational study, we would like to present the overall findings in real-world clinical setting as much as possible. Missing data will be handled using R-MICE package for the following outcome measures (migraine frequency, average pain intensity NRS, MSQ, GAD-7, PHQ-9, ISI) at each evaluation timepoint within the 12 weeks’ observation period.

As mentioned above, this observational study aims to explore the effects of CHM for migraine, summarise patterns of CHM for migraine and the characteristics of migraine patients, we will present what we observed for the patterns of CHM and the characteristics of patients.

The “Analytical methods” section has been revised to clarify this information (Line 244-262).

4. The section also lacks references. The authors refer to the "Apriori Algorithm", but no reference is added with a description of this algorithm

Response:

The reference and brief description have been added as suggested (Line 254-256).

# Comments from reviewer #2

## Introduction

1. I ask the authors to remove the part of the sentence which says that according to the latest classification, the division of migraine in relation to menstruation was made (this statement is not contained in the current classification).

Response:

Thanks for the suggestion. This statement has been removed.

2. Explain what is meant by a poor prognosis in patients with comorbidities.

Response:

An explanation about the “poor prognosis” was added (see Line 57-58).

3. The sentence "Currently there is no cure for migraine" is unnecessary.

List all groups of drugs, and western groups of drugs and western treatments for migraine. It is not enough to list only NSAIDs, triptans, CCB, beta blockers.

Are these drugs also used for comorbidity or just for migraine. If yes, for which comorbidities and provide references that confirm these claims.

Response:

The sentence of "Currently there is no cure for migraine" was deleted.

All groups of western drugs for migraine and the devices are listed in Line 62-71.

Specific drugs which can be adopted for migraine comorbidities as recommended by clinical guidelines were also listed in this paragraph.

## STUDY DESIGN

4. The dose of the medicine that the patients will take be determined on the basis of what references? What previous studies have confirmed the effectiveness of that dose and not some other dose of the drug? On the basis of which studies the administrative methods, courses, forms of CHM will be determined and which previous studies have confirmed that it is the best method, course, form?

Response:

This is an observational study, the treatments for the patients in this study are prescribed by the clinicians according to each patient’ real situation. These interventions are not predefined nor interfered by the observation. We are recording and summarising what we observe in the real-world clinical practice.

5. Is it correct to collect data for patients who were not in the study during the entire planned period?

Response:

This study only collect data from patients who meet the inclusion and exclusion criteria.

## INTERVENTIONS

6. It is necessary to describe in detail the nature of CHM that will be used in this research, to connect it with previous research on animal models or in the laboratory. It is necessary to state the chemical composition of the drug, the chemical formula of the drug, the site of action of the drug, the mechanism of action of the drug, the potential interactions of CHM with drugs for various comorbidities.

Response:

As mentioned above, in this observational study, the CHM interventions are tailored based on each patient’ symptoms and need, and decided by the clinicians, who are independent from the researchers. The CHM treatments to be observed are not able to be anticipated, therefore we are not able to provide the required information in this protocol, but this will be addressed when we report the results of this study. Upon the completion of this observational study, we will identify the most frequent herbs, the core herb pairs and herb combinations being used in clinical practice, and then provide information on their mechanisms for migraine in the final study report.

7. Are these medications used for an acute migraine attack or to prevent an attack or both?

Response:

The western medications and CHM formulations being prescribed by clinicians can be used for either acute migraine attacks or migraine prevention.

## DATA COLLECTION

TREATMENTS, EXAMINATIONS AND DIAGNOSES:

8. Chinese medicine syndrome diagnosis - I would ask the authors to explain the meaning of this term in more detail.

Response:

Chinese medicine syndrome diagnosis is the process of a comprehensive analysis of patients’ symptom and signs, under the principles of Chinese medicine aetiology and pathogenesis. It is used to guide the selection of Chinese medicine treatments.

Based on the Chinese medicine syndrome diagnosis, treatments will be individualised because patients may present different syndromes although they are diagnosed with the same clinical condition; and each patient may present different syndromes in different stages of a whole disease course, therefore the treatment will also be modified accordingly.

## SAMPLING METHOD AND SAMPLE SIZE

9. The number 400 is based on the number of views in which time period? Explain in more detail the selection of this number of patients.

Response:

The estimated number of participants is approximately 400 based on the average annually outpatient visit numbers at the headache clinic of GPHCM between July 2018 and July 2020.

This information has been clarified in the Methods section (see Line 221-222).

End.
